# Construction and verification of the transcriptional regulatory response network of *Streptococcus mutans* upon treatment with the biofilm inhibitor carolacton

**DOI:** 10.1186/1471-2164-15-362

**Published:** 2014-05-12

**Authors:** Padhmanand Sudhakar, Michael Reck, Wei Wang, Feng Q He, Irene W Dobler, An-Ping Zeng

**Affiliations:** Institute of Bioprocess and Biosystems Engineering, Hamburg University of Technology, 21073 Hamburg, Germany; Research Group Microbial Communication, Helmholtz Center for Infection Research, Inhoffenstrasse 7, 38124 Braunschweig, Germany; Luxembourg Centre for Systems Biomedicine, 7, Avenue des Hauts Fourneaux, L-4362 Belval, Luxembourg

**Keywords:** *Streptococcus mutans*, Caries, Biofilm inhibitor, Carolacton, Transcriptome, Reverse engineering, Transcriptional regulatory network, Key regulators, Network verification

## Abstract

**Background:**

Carolacton is a newly identified secondary metabolite causing altered cell morphology and death of *Streptococcus mutans* biofilm cells. To unravel key regulators mediating these effects, the transcriptional regulatory response network of *S. mutans* biofilms upon carolacton treatment was constructed and analyzed. A systems biological approach integrating time-resolved transcriptomic data, reverse engineering, transcription factor binding sites, and experimental validation was carried out.

**Results:**

The co-expression response network constructed from transcriptomic data using the reverse engineering algorithm called the Trend Correlation method consisted of 8284 gene pairs. The regulatory response network inferred by superimposing transcription factor binding site information into the co-expression network comprised 329 putative transcriptional regulatory interactions and could be classified into 27 sub-networks each co-regulated by a transcription factor. These sub-networks were significantly enriched with genes sharing common functions. The regulatory response network displayed global hierarchy and network motifs as observed in model organisms. The sub-networks modulated by the pyrimidine biosynthesis regulator PyrR, the glutamine synthetase repressor GlnR, the cysteine metabolism regulator CysR, global regulators CcpA and CodY and the two component system response regulators VicR and MbrC among others could putatively be related to the physiological effect of carolacton. The predicted interactions from the regulatory network between MbrC, known to be involved in cell envelope stress response, and the *murMN*-SMU_718c genes encoding peptidoglycan biosynthetic enzymes were experimentally confirmed using Electro Mobility Shift Assays. Furthermore, gene deletion mutants of five predicted key regulators from the response networks were constructed and their sensitivities towards carolacton were investigated. Deletion of *cysR*, the node having the highest connectivity among the regulators chosen from the regulatory network, resulted in a mutant which was insensitive to carolacton thus demonstrating not only the essentiality of *cysR* for the response of *S. mutans* biofilms to carolacton but also the relevance of the predicted network.

**Conclusion:**

The network approach used in this study revealed important regulators and interactions as part of the response mechanisms of *S. mutans* biofilm cells to carolacton. It also opens a door for further studies into novel drug targets against streptococci.

**Electronic supplementary material:**

The online version of this article (doi:10.1186/1471-2164-15-362) contains supplementary material, which is available to authorized users.

## Background

*Streptococcus mutans* is an oral pathogen, which along with other closely related streptococci called the mutans streptococci, plays an important role in the formation of caries and tooth decay in humans. This is attributed to its ability to form biofilms which are generally difficult or impossible to eradicate by antibiotic therapy because biofilm cells are resistant to antibiotics [1, 2].

Recently, it was shown that carolacton, a secondary metabolite from the myxobacterial species *Sorangium cellulosum* has a high inhibitory activity against actively growing *S. mutans* biofilm cells resulting in changes in cell morphology, elongation of cell chains, membrane damage and death of a part of the population. Carolacton was also found to induce a dose-dependent damage of *S. mutans* biofilms over a wide concentration range resembling a sigmoid dose–response curve [3]. Carolacton inhibits *S. mutans* biofilms even at nanomolecular concentrations [3] implying that it primarily targets molecular entities which are present only as a few copies per cell. In this regard, carolacton is very similar to compounds which target cellular signaling networks [4] rather than directly targeting particular enzymes in pathways associated with vital processes such as protein, DNA/RNA synthesis, cell division etc.

To decipher the genes whose expression is affected by carolacton, a time resolved transcriptome analysis of *S. mutans* biofilms after carolacton treatment was carried out by Reck et al. [5]. Results from the study indicate that carolacton affects changes in the expression of genes related to biofilm formation, autolysis, pyrimidine and histidine metabolism, cell shape and cell division in addition to two component systems (TCSs). Among the TCSs, the *vicRK* system shows an immediate strong downregulation, while the *comDE* system controlling competence development through quorum sensing [6, 7] is upregulated. A deletion mutant for the histidine kinase encoding gene *vicK* which responds instantaneously to carolacton treatment together with all the *vicR*-coexpressed genes, was similarly tested for carolacton-sensitivity, but since the *vicK* mutant showed very poor biofilm growth, further inferences could not be made. VicR is an essential gene and cannot be deleted in *S. mutans*[8]. Meanwhile, the *S. mutans* eukaryotic-like serine-threonine protein kinase *pknB* whose ortholog has been shown to be a master regulator of virulence in *S. pneumoniae*[9] displayed no observable changes in transcription [5]. However, when *pknB* is knocked out, it results in a carolacton-insensitive mutant [5]. These data show that even though the physiological and genetic responses of carolacton-treated *S. mutans* biofilm cells are known, the underlying network which orchestrates the expression of affected genes in response to carolacton still remains a mystery. This calls for an effort to uncover the effect of carolacton at the network level. Although Reck et al. [5] have already measured the temporal progression of the *S. mutans* transcriptome in response to carolacton, their dataset is characterized by a small number of sampling points (five) and large time intervals, which does not allow for a reliable network inference. Hence, an extended time-series transcriptome is required encompassing a higher number of sampling points with relatively short intervals and was carried out in the current study.

Reverse engineering based network reconstruction methods have widely been used to infer genetic networks from gene expression data measured most commonly using cDNA microarrays. Excellent reviews about genetic network reconstruction from expression data have been published [10]. In order to capture the network level events of biological systems upon exposure to various stimuli, reverse engineering methods have been developed to infer underlying networks from time series expression data in addition to static data [11]. For instance, methods such as Time Series Network Identification (TSNI) were used for inferring co-expression networks and compound mode of action from time-series gene expression data following interventions [12]. Although the inferred co-expression network using TSNI involved only nine genes of the *E. coli* DNA-damage response pathway, it is seen as one of the first attempts in network reconstruction for determining the compound mode of action from a time series transcriptome. Further tools and algorithms were also developed to deal with whole genome network inference [13, 14], but the gene-gene interactions in such inferred networks were either undirected (no assigned causality) or carry only a statistical probability of causality. Despite lacking directionality, undirected co-expression networks have nevertheless been used to infer critical genes and networks involved in T cell functions [15].

Meanwhile, whole genome directed networks were inferred by using combinatorial methodologies [16] incorporating gene-gene interactions from reverse engineering algorithms with biological information such as data on binding motifs and promoter elements [17], functional genomics [18], genome annotation [19] and transcription factor activities [20]. Although non-exhaustive due to the lack of sufficient biological information, genome wide transcriptional regulatory networks have nevertheless been constructed from heterogeneous data for a few organisms such as *Escherichia coli*[20], *Saccharomyces cerevisiae*[21], *Bacillus subtilis*[17], *Candida albicans*[22], *Streptomyces coelicolor*[18], *Halobacterium NRC-1*[19], *Pseudomonas aeruginosa*[23] and others. Despite the advances made in the field of genome-wide regulatory network reconstruction, most of these attempts have been carried out for model or standard organisms (for which ample biological information exists) such as *E. coli, S. cerevisiae, B. subtilis, P. aeruginosa*[17, 20, 23, 24]. Some progress has also been made in the case of human cells as shown by Basso et al. where human B cell gene regulatory interactions were inferred [25]. Hence, one of the major current challenges in the field is the network inference for less-studied organisms which are either pathogens or are industrially important.

Even though genetic networks have been inferred under drug treatment conditions for some organisms, most of them have either been limited to compounds and molecules whose targets and mode of action were already known and/or limited to model organisms and certain human cell lines. To the best of our knowledge, reconstruction of a contextual genome-scale regulatory network for the human caries pathogen *S. mutans* has not yet been carried out although studies focusing on genome-wide transcriptome profiling have been reported [5, 26–29]. A workflow based on a combinatorial strategy was executed in an attempt to infer a genome-wide network for *S. mutans* biofilms under conditions of treatment with the biofilm inhibitor carolacton. The Trend Correlation method which has been used to construct co-expression networks of human T cells [15] was used for inferring the *S. mutans* co-expression network under carolacton treatment conditions using microarray data generated in this study. This was followed by the construction of a transcriptional regulatory response network (TRRN) by incorporating binding site information. From the TRRN, we detected sub-networks whose activation or repression could be related to the biological effect of carolacton. A key regulator was identified based on its connectivity in the regulatory network and when knocked out, rendered *S. mutans* biofilms insensitive to carolacton. Furthermore, tested interactions from the inferred regulatory response network were confirmed using *in vitro* interaction detection assays.

## Results and discussion

### Dynamic genome-wide transcriptome profiling

A high resolution transcriptomic time-course experiment with *S. mutans* biofilms (with 11 samples taken from 5 to 300 min post carolacton treatment) was performed to capture the temporal response over an extended period of time. Our time-series data included results from a sample taken at 5 min post carolacton treatment and is expected to identify genes whose expression patterns were immediately affected by carolacton. A total of 772 GTAAs (genes corresponding to transcripts with altered abundances) or differentially expressed genes were identified at a log-fold change > =0.8.

Enrichment analysis with respect to various categories such as biological functional classes and KEGG metabolic pathways was performed for the up- and downregulated GTAAs at every time point in order to gain an overview of the various categories of genes affected by carolacton (Additional file 1). The results from the enrichment analysis of upregulated GTAAs indicated that transcripts from the pyrimidine ribonucleotide biosynthesis (P-value < = 4.09E-18), nucleosides, purines and pyrimidines’ transport (P-value < = 2.45E-2), and metabolism of alanine, aspartate and glutamate (P-value < = 7.2E-4) were most significantly over-represented at both 5 min and 20 min but not at 40 min. These observations suggest a possible specific role of pyrimidine biosynthesis and metabolism in the initial response of *S. mutans* biofilms to carolacton. At later time points however, many more categories were found to be over-represented among the set of upregulated GTAAs due to probable cascade effects.

Enrichment analysis of the downregulated GTAAs at 20 min revealed categories such as glutamate biosynthesis, TCA cycle, anaerobic energy metabolism, glyoxylate and dicarboxylate metabolism, nitrogen metabolism as well the signal transduction related two component systems (TCSs). TCSs impart various virulence characteristics to *S. mutans* by sensing and responding to various signals related to stress, thus enabling it to survive and tolerate unfavourable conditions [30, 31]. The transcriptional downregulation of TCS genes could indicate a possible effect of carolacton having a repressive effect on the signaling mechanisms of *S. mutans*. In general, downregulated GTAAs in the early part of the response were enriched with categories corresponding to the processes of signaling, energy and amino acid metabolism. The set of downregulated GTAAs in the later phases after 20 min was also found to be enriched with many categories over the course of the experiment. Interestingly, the alanine, aspartate and glutamate metabolic pathways were found to be over-represented in both the up- and downregulated GTAA sets at the time point of 20 min after carolacton treatment suggesting a dual regulation of such pathway enzymes in the initial response to carolacton. The information from our transcriptome data largely agrees with those from a previous study by Reck et al. [5] where only five sampling points with large intervals were used.

### Contextual co-expression network

**Figure 1:**
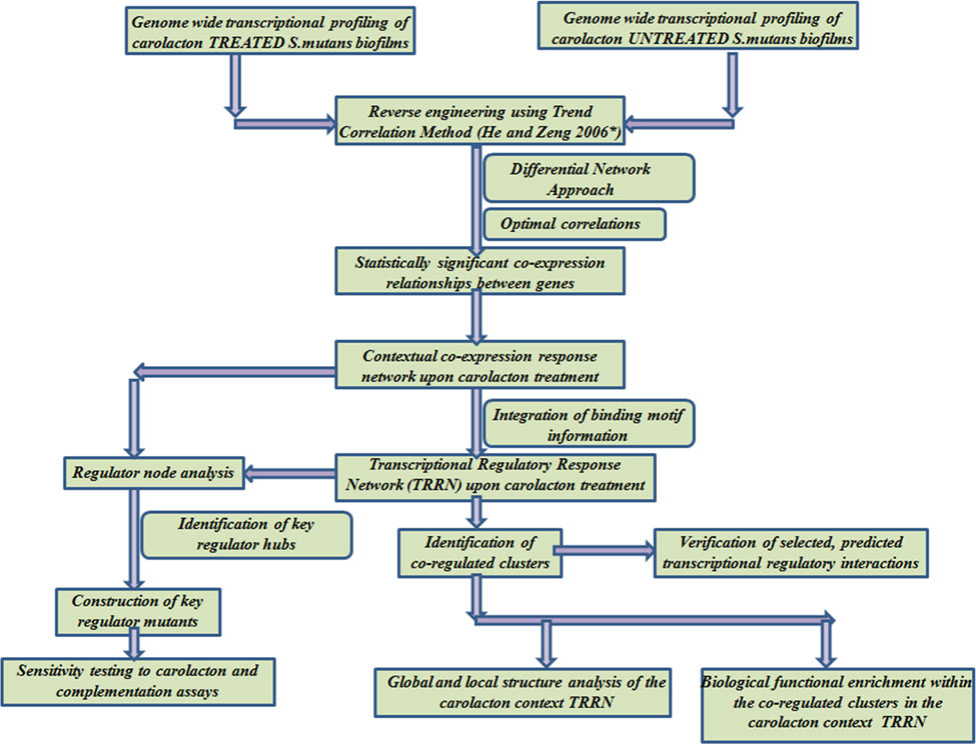
**Workflow to capture the network level effects of the biofilm inhibitor carolacton on** ***S. mutans*** **biofilms.** The directions of the arrow marks denote the flow of data processing and sequential steps. Shapes of boxes have no particular significance while the descriptions within the boxes represent the steps corresponding to data generation, algorithms, data processing, network and experimental analyses. *indicates the reference [32].

In order to identify co-expression relationships among genes immediately affected by carolacton, the co-expression network was confined to statistically significant optimal correlations which started either from 0, 5 or 20 min after carolacton treatment (see Methods). The contextual co-expression network inferred according to the workflow shown in Figure 1 consisted of 8284 gene-gene co-expression relationships (see Additional file 2). 5430 (65.5%) of the 8284 edges were characterized by time lagged co-expression relationships. 3959 (47.7%) of the total number of co-expression relationships could be described as being inverted (opposite change trend in expression patterns) whereas the remaining were described as being positive (showing similar change trends).

### Regulator-target gene binding site map of *S. mutans* UA159

The edges of the carolacton-context co-expression network derived from the dynamic transcriptome data are undirected. In other words, the mere presence of an edge between two genes does not necessarily represent a transcriptional regulatory relationship between them. Although time-lags between the co-expressed regulator and target gene(s) could possibly serve as a preliminary proof of direct/indirect transcriptional regulation [10], further evidence is required for at least assigning putative biological causality and for pursuing precise experimental validation of transcriptional regulatory relationships.

Among the more than 100 known or predicted transcriptional regulators in the *S. mutans* UA159 genome, only a few have been studied experimentally and characterized with respect to their DNA binding site(s). We compensated for the non-availability of experimentally verified transcriptional regulatory binding sites for the *S. mutans* transcriptional factors by applying comparative genomic approaches [33–35]. Binding site data for 44 transcriptional regulators were recovered from different sources such as experimental data from *S. mutans* itself or information from comparative genomic analysis. In cases where the collective number of binding sites inferred for a transcriptional regulator were fewer or equal to three, respective regulogic [33] sites from other species within the Streptococccus genus were retrieved. These were then used to construct PSSMs (Position Specific Scoring Matrices, see Methods) with improved predictive capacities. PSSMs were also constructed from regulogic sites available in the RegPrecise database [36] and from phylogenetic footprinting studies. An online Regulatory Sequence Analysis Tool (RSAT) termed *matrix-quality*[37] was used to objectively determine the optimal P-value for every PSSM (see Additional file 3). The PSSMs were then used to predict binding sites (at the corresponding optimal P-values) in the upstream regulatory regions of all the genes in the *S. mutans* UA159 genome. A total of 1397 unique regulator-target gene relationships (corresponding to 2056 predicted binding sites) based on binding site data alone corresponding to 44 *S. mutans* UA159 transcriptional regulators were thus compiled (see Additional file 4). After integrating operon structure information, 2445 regulator-target gene relationships were found to comprise the final regulator-target gene binding site map. The global regulators CcpA (catabolite repressor protein) and CodY (global regulator of amino acid metabolism) together accounted for ~29.22% of the 2445 regulator-target gene relationships based on binding sites.

### *S. mutans* Transcriptional Regulatory Response Network (TRRN) under carolacton treatment

**Figure 2:**
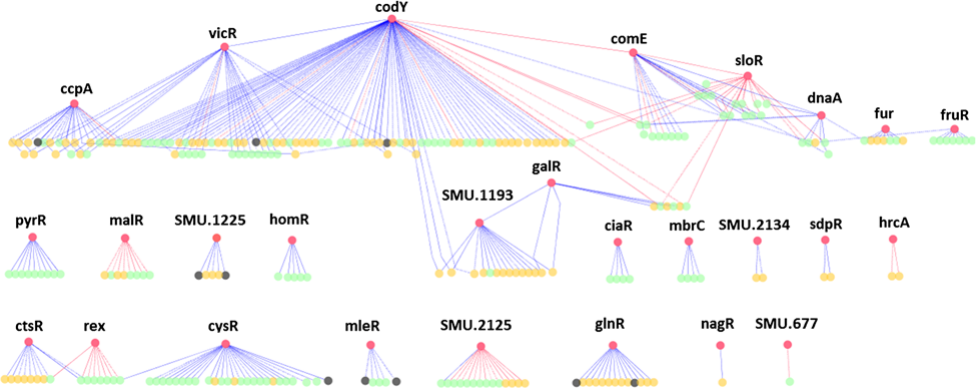
**Topological view of the transcriptional regulatory response network (TRRN) of** ***S. mutans*** **biofilms upon carolacton treatment.** The TRRN was inferred by overlaying the regulator-target gene binding site map onto the co-expression network. It consisted of 27 co-regulated gene groups or subnetworks each under the control of a transcription factor and comprised 329 regulatory interactions involving 307 genes. Some of the co-regulated gene groups overlap with each other as a result of genes modulated by more than one transcription factor. The 27 regulators with outgoing connections (marked with red circles) along with 10 other regulators only with incoming connections (marked with black circles) found to be among target genes within the sub-networks are indicated as well as non-regulator target genes (indicated by green circles). If the upstream regulatory regions of the target genes or their corresponding operons harbored multiple putative binding sites of its predicted regulator(s), then they are indicated by orange circles). In addition, the regulators with outgoing connections are also marked with their gene names above their respective nodes. Blue arrows indicate a positive (activation) relationship whereas red arrows stand for a negative (repression) effect. Connections always flow from top to bottom. Spatial positions of transcription factor nodes are manipulated so as to pictorially depict the possible hierarchies. The carolacton context TRRN shown herein is observed to be organized as a double layered hierarchy. The network was visualized using Cytoscape.

Combining inferred correlation relationships from the co-expression network and the regulator-binding site map resulted in 227 transcriptional regulatory relationships with predicted direct biological causality (see Additional file 5). After operon oriented adjustment (see Methods), the transcriptional response regulatory network (TRRN) (Figure 2) comprised 329 relationships among 307 genes, of which 37 were found to be transcription factors. Based on the predicted transcriptional regulatory connections, the TRRN genes could be organized into 27 co-regulated gene groups or sub-networks. A co-regulated gene group is defined as a set of genes which are putatively regulated by a common transcription factor. 10 of the 37 regulators were associated with predicted incoming connections only and were contained within the 27 sub-networks with different size distributions. The largest sub-network was that putatively modulated by CodY (which is a known global regulator [38]) and comprised 84 genes. The second largest sub-network consisted of 26 genes under the putative control of CysR (the cysteine metabolism regulatory protein encoded by SMU_852) followed by two sub-networks each containing 22 genes and predicted to be modulated by the essential TCS response regulator VicR and the global regulator CcpA, respectively.

The TRRN (Figure 2) represented a “waterfall-like” model with respect to its global topological structure and hierarchy [39, 40] with some regulators apart from modulating their own sub-networks also exerting indirect control over other sub-networks by putatively modulating the expression of the corresponding transcription factors. For example, it can be seen from the inferred TRRN (Figure 2) that the global regulator CodY putatively regulated the expression of two distinct genes encoding the TCS response regulators VicR and ComE, thus potentially exerting an indirect influence on their sub-networks as well. Although global hierarchical organization was observed in the inferred TRRN, it was nevertheless confined only to a double-layered hierarchy compared to multi-layered hierarchies reported in the regulatory networks of well-studied organisms [40, 41]. This could primarily be due to the lack of biological connectivity data, firstly as a result of the limited experimentally verified binding site information of *S. mutans* transcription factors. Secondly, the functional and/or evolutionary divergence of *S. mutans* transcription factors from their corresponding orthologs in standard organisms has a certain limiting influence on the comparative genomic based extrapolation of binding sites [33].

The overlap between co-regulated gene groups, which was previously observed in bacteria [42, 43], is also a characteristic of the inferred TRRN. 13% of the 307 TRRN genes were found to be under the putative control of more than one transcriptional regulator (implying an incoming degree > 1). From a local network structure point of view, the nodes putatively controlled by just one regulator resemble single input motifs (SIM) whereas the nodes co-regulated by several regulators form multiple input motifs (MIMs) [44]. Besides MIMs, overlap between co-regulated gene groups could be the manifestations of more complicated network motifs such as the feed forward loop (FFL) in which a regulator encoding gene is modulated by another regulator, both of which control the expression of a common gene [45]. If multiple genes are controlled by a single FFL, then it is termed as a multi-output FFL (MOFFL) [46]. Additional file 6 lists the various types of significant motifs identified (P-value < = 0.01 after 10000 randomizations) in the carolacton context dependent TRRN among which were found FFLs including other motifs such as regulatory bi-fans [44] and their derivatives [47]. Local network topologies such as the motifs found here were consistent with previous observations in other organisms [44–46, 48] and could be important for eliciting quick and flexible responses to stimuli [45]. Although the exact biological mechanisms which give rise to such motifs remain to be understood, these observations demonstrate not only the relevance of the constructed network but can also be further used for detailed dynamic quantitative modeling of smaller sub-systems.

### Biological context within co-regulated sub-networks in the TRRN

**Figure 3:**
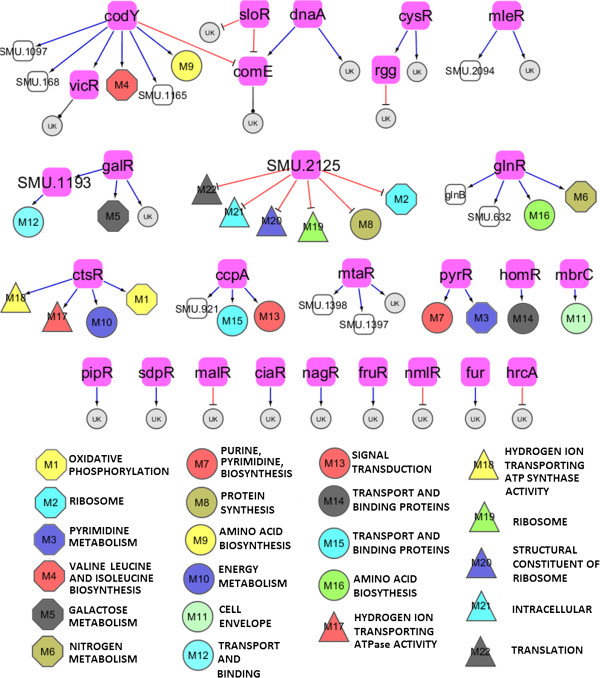
**Categorical enrichment within the sub-networks comprising the** ***S. mutans*** **biofilms TRRN upon carolacton treatment.** The co-regulated groups or sub-networks within the TRRN were found to be enriched with functional categories such as KEGG metabolic pathways, biological functional classes and gene ontology terms thus lending biological meaning to the inferred regulatory response network. Co-regulated groups denote sets of genes predicted to be commonly modulated by a transcription factor (indicated by filled pink rectangles). The regulatory response network also includes other transcriptional factors with only incoming connections (black rectangles in bold) and found to be among the co-regulated groups. Some of the co-regulated groups were found to be enriched with functional categories such as KEGG metabolic pathways (octagons), biological functional classes (circles) and gene ontology terms (triangles) whereas others (black circles filled in with letters ‘UK’) did not display any enrichment. Black edges with ellipsoid target edges represent relationships with dual regulation (some of the transcriptional regulatory relationships within the sub-network are characterized by positive (activational) expression patterns and others by inverted (repressive) expression patterns between the regulator and the target gene). The meanings of all the other arrow symbols are as described in the legend of Figure 2.

The 27 co-regulated groups or sub-networks contained statistically significant over-represented (significance score > =0) functional categories such as biological functional classes, KEGG metabolic pathways and gene ontology terms (see Additional file 7). Figure 3 visualizes the TRRN enrichment data shown in Additional file 7. Put together, 22 enrichment events involving 6 KEGG metabolic pathways, 7 biological functional classes and 6 gene ontology terms were collectively identified within the 27 sub-networks. At least 7 cases were found in which the transcription factor or regulator belonged to the same functional category as that found to be over-represented in its target gene group, confirming that the constructed regulatory network is biologically meaningful. For instance, the sub-network co-regulated by the glutamine synthetase repressor GlnR known to control glutamine metabolism [49] in *S. mutans* was enriched with genes belonging to the functional categories “amino acid biosynthesis” and “nitrogen metabolism”. On closer observation, most of the genes within the GlnR co-regulated sub-network were related to glutamine metabolism. Genes belonging to the biological functional class of cell envelope metabolism were over-represented in the sub-network co-regulated by the TCS RR MbrC associated with cell envelope stress response [50]. Similarly, the genes putatively modulated by the pyrimidine regulatory protein PyrR contained an over-representation of pyrimidine metabolism genes. Several instances were also observed in which the functional category to which the regulator belonged to was different from that found to be over represented within the given sub-network (Figure 3, Additional file 7). Besides the above described intra- and inter categorical relationships between regulators and their sub-networks, the TRRN also contained regulatory relationships which were already verified by others (see Additional file 5).

### TRRN sub-networks co-regulated by global transcription factors

The sub-networks of the global transcriptional regulators affecting central metabolism namely CodY and CcpA [38, 51] were among the top three sub-networks in terms of the number of genes. CcpA is reported to modulate sugar uptake and metabolism, carbon catabolite repression and expression of virulence related genes in *S. mutans*[51]. The treatment of *S. mutans* biofilms with carolacton caused an immediate downregulation of CcpA modulated genes (Additional file 8). The CcpA sub-network consisted of genes encoding components of two mannose specific phosphotransferase systems (*ptnAC (manLM)*-SMU_1879 and SMU_1960c-SMU_1961c) [52–54], as well as three ATP dependent transporter complexes (*msmEFG*, SMU_921-SMU_922 and SMU_1315c-SMU_1317c) among others. While *msmEFG* is reportedly involved in the energy dependent transport of multiple sugars [55], the substrate specificities of the other two clusters are unknown. Sub-networks of other sugar-specific transcription factors such as the repressors GalR and FruR of galactose and fructose metabolism respectively were also found to be downregulated. Sugars serve as substrates for the synthesis of glucans and associated by-products required for adherence to the tooth surface, biofilm formation as well as for the formation of organic acids which aid cariogenic disease progression [56–60]. The sub-network co-regulated by CodY (Additional file 8) meanwhile was found to be enriched with genes belonging to the metabolism and biosynthesis of amino acids, particularly the branched chain ones such as leucine, valine and isoleucine. The control of the metabolism of these amino acids by CodY has already been reported [38]. Hence, from the preliminary analysis of the regulatory response network, it can be stated that carolacton has an inhibitory effect on the central metabolism of sugars and amino acids by downregulating genes co-regulated by the global transcriptional factors CcpA and CodY.

### Immediate induction of the pyrimidine metabolism

**Figure 4:**
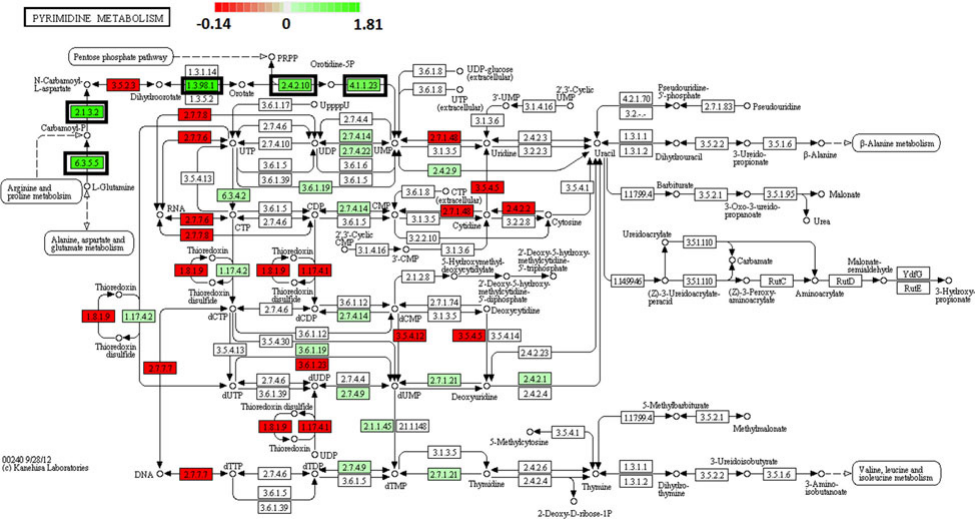
**Heat map representation of the transcriptional response of the** ***S. mutans*** **pyrimidine metabolic pathway upon carolacton treatment.** Genes from the pyrimidine metabolism pathway were among the first to be modulated upon carolacton treatment. The log2-fold expression change of pathway genes at 5 min post treatment were used for the heat-map representation. Green indicates upregulation and red downregulation. The scale is indicative of the corresponding changes in normalized gene expression. Pathway genes encoding enzymes catalyzing reactions leading up to UMP were strongly upregulated while most of the other pathway genes exhibited relatively weak modulation. Enzymes marked in black bold rectangles indicate the corresponding strongly upregulated transcripts of the pathway. White cells correspond to pathway enzymes not found in the genome of *S. mutans* UA159. If a particular enzyme corresponds to multiple transcripts (as a result of multiple protein subunits constituting an enzyme), then the transcript with the highest amplitude of log2-fold change was used. Graph generated using the Mayday visualization tool version 2.12.

**Figure 5:**
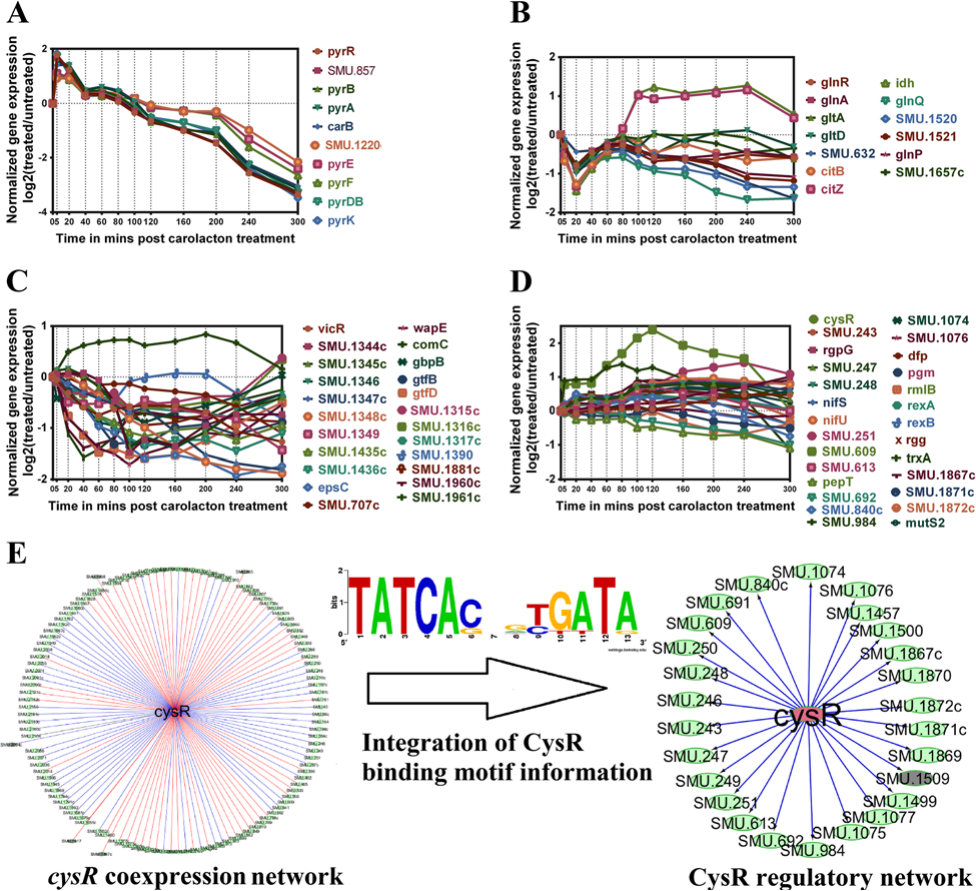
**Normalized expression profiles of genes modulated by selected transcription factors in response to carolacton treatment. (A)** The normalized expression profiles of the genes co-regulated by the pyrimidine biosynthesis regulatory protein in the carolacton treatment context TRRN of *S. mutans*. Two operons containing genes encoding enzymes involved in the biosynthesis of pyrimidine ribonucleotides were upregulated sharply by about 1.8 to 2 log2-fold 5 min post carolacton treatment. **(B)** The expression dynamics of the genes co-regulated by the glutamine repressor GlnR in response to carolacton treatment. **(C)** The normalized expression profiles of the genes commonly regulated by the downregulated essential TCS response regulator *vicR* in response to carolacton. VicR modulated genes include those encoding virulence attributing products such as glucosyltransferases B and D, cell wall protein WapE among others. **(D)** Temporal behavior of the 26 genes found within the co-regulated group/subnetwork commonly modulated by SMU.852 encoding the CysR cysteine metabolism regulatory protein. Co-regulated gene groups were constructed by overlaying predicted regulator-binding site maps onto the co-expression network as shown here **(E)** specifically for the network confined to *cysR*. The node corresponding to the lone regulator (SMU.1509 encoding a putative Rgg family transcription factor) in the gene group co-regulated by CysR is marked in grey.

Peptidoglycan is an important constituent of the gram positive cell-wall. It is expected that cell membrane damage and biofilm inhibition would have a substantial effect on pathways and genes related to cell wall synthesis and metabolism. UDP-N-acetylglucosamine, a key intermediate in the biosynthetic process of the cell wall component peptidoglycan, is produced by glycolysis, sugar metabolism as well as the pyrimidine metabolic pathway [61]. Expression data indicate the absence of immediate modulation and at later time points the downregulation of the glycolytic pathway as well as of the pathways related to the metabolism of various sugars such as fructose, mannose and galactose. On the contrary, two pyrimidine biosynthesis gene clusters (namely the *pyrEFDZ* and *pyrRPBA-carB* operons) belonging to the PyrR sub-network and coding for the enzymes of the pyrimidine metabolism pathway (Figure 4) were upregulated by about 1 to 1.8 log2-fold at 5 min post treatment (Figure 5A). It is of note that most of the genes in the pyrimidine metabolism pathway were not transcriptionally altered with the exception of the two strongly upregulated *pyrEFDZ* and *pyrRPBA-carB* operons. These operons encode enzymes catalyzing the biochemical steps leading to the production of UMP and UDP (see Figure 4) suggesting that this part of the pathway is specifically activated. An upregulation of the pyrimidine metabolism pathway would produce pools of UDP-N-acetlyglucosamine (UDP-N-AG) for peptidoglycan synthesis compensating carolacton-induced membrane and cell wall damage. The upregulation of pyrimidine biosynthetic steps was also observed in an *S.aureus* strain harboring a mutation of a two component system essential for cell wall metabolism [62].

### Carolacton affects glutamine metabolism

Genes in the TRRN predicted to be co-regulated by the glutamine synthetase repressor GlnR along with *glnR* itself were associated with a sharp downregulation initially (Figure 5B). It is known that glutamine is an important source of nitrogen for *S. mutans*[63]. Some of the GlnR-modulated genes in response to carolacton treatment included a glutamine transporter gene-cluster (SMU_1519-SMU_1522), the glutamine/glutamate biosynthesis operon (*glnRA-gltAD*), as well as an operon consisting of the putative ammonium transporter coding gene *nrgA* and the nitrogen regulatory protein coding gene *glnB*. Other glutamate and glutamine metabolism related genes such as *citZH* and *idh*[64], whose modulation was also reported to be mediated by GlnR [49], were downregulated initially until 20 min post carolacton addition. The protein products of the *citZH* and *idh* genes catalyze the steps leading to the formation of alpha-ketoglutarate from oxaloacetate (feeding the de-novo glutamine synthesis pathway) [64]. The expression pattern of the genes predicted to be controlled by GlnR suggests an activational (positive) rather than an inverted (negative) relationship. But GlnR is known to function as a repressor protein. However, this does not seem to be a conflicting observation since GlnR orthologs have been known to have dual activator-repressor functions [65]. Hence, the *S. mutans* GlnR could also be a dual regulator given that several transcriptional regulators with dual functions have been reported earlier in *S. mutans*[66–68].

Glutamate is known to play a role in the acid tolerance response (ATR) mechanism of *S. mutans*[69–71] which is crucial for the survival and adaptation of *S. mutans*. Carolacton has the maximum effect in terms of membrane damage and cell death in actively growing biofilm cultures at low pH. But the transcriptional changes (immediate downregulation until 20 min post treatment) of *glnR* and its target genes occurred at a neutral pH (data not shown) thus ruling out ATR as a cause for the intial modulation of GlnR mediated genes. At later time points which correspond to a low pH, the upregulation of the GlnR-modulated genes occurred although a downregulation should have been the expected trend to enhance the ATR. This suggests that carolacton directly or indirectly diminishes the ATR thereafter. Alternatively, the upregulation of the glutamine metabolism genes comprising the GlnR co-regulated gene group after the initial downregulation could possibly be explained as a means to provide precursors for peptidoglycan biosynthesis. Thus, it is plausible that the pyrimidine metabolism is induced to feed the peptidoglycan biosynthesis during the initial phase (until 20 min after carolacton treatment) followed by the upregulation of the glutamine metabolism related genes during the later phase.

### TRRN genes co-regulated by the essential response regulator VicR

The microarray data show that *vicKR*, one of the earliest responding TCSs upon carolacton treatment, was downregulated. The *vicKR* TCS plays a major role in the biofilm formation, competence development, oxidative stress tolerance, acid tolerance, autolysin production, glucan and fructan metabolism of *S. mutans*[27, 72–77]. In addition, the response regulator *vicR* has also been identified as being essential for the survival of *S. mutans,* i.e. it is an essential gene which cannot be deleted [8]. The gene group co-regulated by VicR (Figure 5C) in the TRRN consisted mostly of downregulated genes including those encoding surface structure and cell wall proteins such as glucosyltransferases (gtf) B and D, glucan binding protein B (gbpB), and the sortase-anchored cell wall protein *wapE*[78] among others. This is consistent with the previous study [5] whereby all the genes including the ones described here and co-expressed with the downregulated *vicR* had reduced transcript abundances. The evidence put together suggests that carolacton could interfere with the signalling mechanisms related to the activity of this essential response regulator thereby exerting its biofilm inhibitory and membrane damaging effects. Some of the genes such as *gtfB*, *gbpB* and *comC* in the VicR co-regulated gene cluster were already known to be direct transcriptional regulatory targets [76, 79, 80] of the VicR protein thus contributing to an independent verification of our network prediction based on time-series microarray data and binding motif information.

### Modulation of genes co-regulated by the cysteine metabolism controlling transcription factor CysR

Genes connected by edges to *cysR* (encoding a regulatory protein controlling the metabolism of sulfur containing amino acids [81]) in the co-expression network had both inverted and positive expression patterns compared to that of *cysR* (Additional file 2). After combining the CysR binding motif information [81], directionality was assigned to the edges after which all of the *cysR* co-regulated genes were having only positive expression patterns (Figure 5D) in relation to *cysR*. CysR was found to modulate 26 TRRN genes (Figure 5E) making it the transcriptional regulator with the second highest degree (number of modulated genes) in the carolacton context TRRN. Some of the genes transcriptionally modulated by CysR include SMU_609 encoding a putative-40 K cell wall protein precursor, SMU_246 encoding a putative glycosyltransferase-N-acetylglucosaminyltransferase and SMU_984 whose gene product has been predicted to be an uncharacterized autolysin among others. Besides, SMU_1509 whose gene product is a Rgg family [82] regulator protein was also observed to be among the CysR sub-network as were genes encoding putative Nif proteins related to nitrogen metabolism and those such as the SMU_1074-SMU_1077 operon involved in cysteine metabolism. Statistically significant enrichment for various functional categories was not observed within the CysR sub-network and this might be attributed to the low number of genes from any given category. Nevertheless, the functional relevance of the individual genes (such as SMU_609 which is one of the strongest modulated genes with > 2 log2-fold upregulation in response to carolacton treatment) within the CysR sub-network as well as its connectivity in the TRRN point out the importance of CysR in the response of *S. mutans* biofilms to carolacton.

### Experimental verification of the predicted regulatory interactions between MbrC (BceR) and the *murMN*-SMU_718 operon

By combining gene expression and binding motif information, *mbrC* was predicted to regulate four so far unidentified target genes (the SMU_716-SMU_717-SMU_718c operon and SMU_610 encoding the cell surface antigen SpaP) in the carolacton treatment context TRRN. The autoregulatory response regulator MbrC -also known as BceR- is encoded within a four-gene operon (*mbrABCD*/*bceABRS*) and has been shown to regulate cell envelope stress response mechanisms in *S. mutans*[50]. Ouyang et al. demonstrated that MbrC transcriptionally regulates the genes SMU_302, SMU_862, SMU_1006 and SMU_1856 by binding to their promoter elements via a conserved binding motif. The binding motif consensus consists of a conserved pair of inverted repeats separated by 2 variable nucleotides (TTACAAnnTTGTAA) [50].

**Figure 6:**
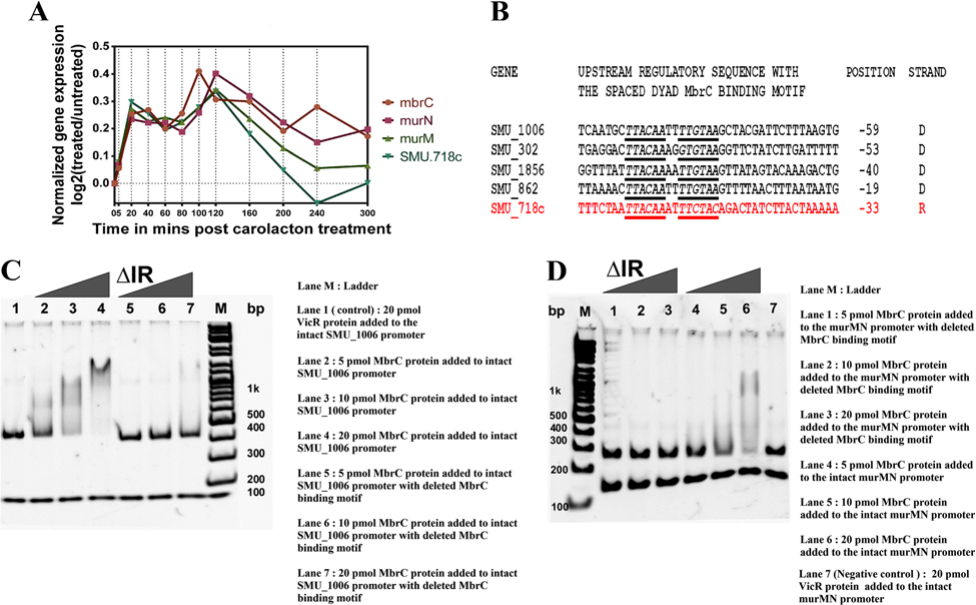
**Experimental verification of the predicted transcriptional regulation of the** ***murMN-*** **SMU_718c operon by the response regulator MbrC.** The predicted transcriptional regulatory relationship was based on a well correlated **(A)** expression profile between *mbrC* and the *murMN*-SMU_718 operon as well as the presence of a **(B)** putative MbrC binding site (TTACAA-AT-TTCTAC) in the upstream regulatory regions of the *murMN*-SMU_718 operon. The alignment among the MbrC binding sites in other experimentally verified targets (black) reported by Ouyang et al. [50] and the putative site upstream of the predicted target (red) *murMN*-SMU_718 operon is shown. The signature repeats of the MbrC binding motif are italicized, underlined and shown in bold. **(C)** Binding of MbrC to the promoter region of the gene SMU_1006 (positive control) was verified using Electro Mobility Shift Assays (EMSA), as already reported by Ouyang *et al.*[50]. **(D)** EMSA also provided the verification of the in-vitro binding of the MbrC protein to the promoter region of the predicted target *murMN*-SMU_718c operon via the putative binding site thus confirming that the latter is a transcriptional regulatory target of MbrC. The triangles indicate increasing concentrations of MbrC in the binding reactions. Black triangles followed by IR indicate target DNA fragments lacking the MbrC binding site.

Among our predicted MbrC target genes, SMU_718c codes for a hypothetical protein with a haloacid dehalogenase-like domain, and SMU_716 and SMU_717 encode two different enzymes, MurN and MurM respectively. These enzymes catalyze the last steps of the peptidoglycan biosynthesis pathway and also play an important role in imparting resistance to cell wall-acting antibiotics [83–86]. Figures 6A and B illustrate the coexpression of *mbrC* with the *murMN*-SMU_718c operon genes and the presence of the potential MbrC binding site (TTACAA-AT-TTCTAC) upstream of the putative target *murMN*-SMU_718c operon respectively. This potential binding site differs from the motif consensus identified by Ouyang et al. [50] by the presence of two substitutions in the inverted repeat and is located upstream (between −33 and −20) of the transcriptional start site of the *murMN*-SMU_718c operon.

To verify this predicted regulatory relationship, Electro Mobility Shift Assays (EMSA) were carried out. The promoter region of SMU_1006 served as a positive control as the binding of MbrC to it was reported previously [50]. An unrelated DNA fragment (around 100–150 bp, lower DNA band in all the lanes) was added in all the samples to prove the specificity of the binding reaction. As shown in Figure 6C, the results confirmed the binding of MbrC to the SMU_1006 promoter as well as the necessity of the consensus motif for the binding of MbrC to its target. When an unrelated transcriptional regulator VicR was used instead of MbrC in the binding reaction as a control to test the specificity of the assay, no shifting of the DNA band was found while with increasing MbrC concentrations, a clear shift was observed (upper band around 350 bp). No significant shift was observed upon deleting the binding motif from the SMU_1006 promoter. Similarly, MbrC also bound to the promoter region of SMU_718c as indicated by a band shift at MbrC concentrations higher than or equal to 5pmol (Figure 6D). With increasing amounts of MbrC (5–20 pmol) present in the binding reaction mixture, a shift from 250 bp (upper DNA band) to around 1 kbp was observed. No shift was observed in the control lane corresponding to the reaction mixture containing an unrelated transcriptional regulator VicR (whose binding site was not present in the *murMN*-SMU_718 promoter) confirming the absence of non-specific binding. Furthermore, the deletion of the MbrC binding site from the *murMN*-SMU_718 promoter also abolished the binding of MbrC to its DNA target. Thus the observations from the EMSA studies have confirmed that the *murMN*-SMU_718 operon is a direct target of the MbrC response regulator as predicted from the regulatory response network. The two substitutions present in the MbrC binding site upstream of the *murMN*-SMU_718c operon could reflect a less stringent binding of MbrC. Accordingly, the shift of the SMU_718c target DNA occurred at a higher MbrC protein concentration (10 pmol) than that (5 pmol) observed for the corresponding SMU_1006 target DNA.

Our finding that MbrC directly controls the expression of two enzymes involved in a central step of cell wall synthesis further highlights the importance of this response regulator for the cell envelope stress response and for maintaining cell wall homeostasis. The reconstitution of the Gram positive bacterial cell wall during growth is intricately linked to the synthesis of peptidoglycan polymer chains [87]. Both *murM and murN* encode alanine adding enzymes involved in the branched peptide peptidoglycan biosynthetic pathway and have also been reported to be primary determinants of the peptidoglycan stem peptide structure [83, 86] which in turn affects various virulence properties such as antibiotic resistance [88–90] and pathogenesis [91]. Thus, although the transcriptional induction of the *murMN*-SMU_718 operon and the *mbrC* gene is relatively weak following carolacton treatment, it might still be significant since only a few copies of the enzymes might potentially be required to catalyze the peptidoglycan biosynthetic reactions. Post transcriptional regulation of enzymes involved in cell wall synthesis and cell division might also significantly contribute to a quick and efficient adaptation of the cell to maintain cell wall homeostasis. Thus small transcriptional changes might result in strong changes of enzyme activity. Induction of the MbrC regulon by carolacton is reminiscent of the previously observed [50] bacitracin induced cell envelope stress response mediated by this regulon in *S. mutans*. This finding further points out the influence of carolacton on cell wall synthesis as reported previously [5].

### Deletion mutants of “key” transcriptional regulators

Based on the inferred TRRN and co-expression networks, single gene deletion mutants corresponding to five different transcriptional regulators (CysR, Rgg, GlnR, SpxA, and FabT) were constructed. We chose these potential key genes for experimental evaluation based on the following criteria. Next to that of CodY, CysR (SMU_852) was identified as the regulator with the highest connectivity in the regulatory response network. CodY is a well-known global master regulator [38] and its deletion has been shown to have obvious phenotypic effects such as the reduced capacity to form biofilms [38]. Since biofilm growth is a pre-requisite for the inhibitory action and membrane damage caused by carolacton, *codY* was not chosen as a deletion candidate. *rgg* (SMU_1509) was identified as the only transcriptional regulator encoding gene among the CysR co-regulated genes and hence was chosen as a candidate for deletion. Alterations in the cell wall metabolism could play a critical role in the response of *S. mutans* biofilms to carolacton due to the observed membrane and cell wall damage [3, 5]. GlnR was chosen as a possible knock-out target due to its importance in peptidoglycan biosynthesis and cell wall metabolism via many of the glutamine-metabolism and transport genes which it modulates as observed in the TRRN. *spxA* (SMU_1142) and *fabT* (SMU_1745) were identified as the top two nodes purely based on connectivity in the co-expression network and hence chosen for further testing. The other genes from the co-expression network were not chosen in this work.

**Figure 7:**
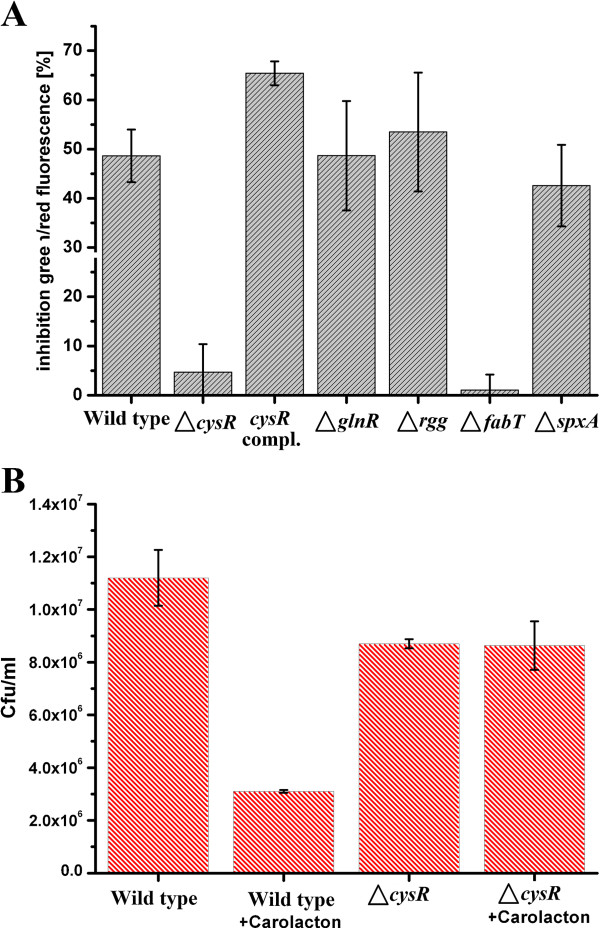
**Effect of deleting five “key” transcriptional regulators and sensitivity of the** ***cysR*** **deletion mutant to carolacton treatment. (A)** Inhibition of viability caused by carolacton treatment was tested for the biofilms of gene deletion mutants of 5 key transcriptional regulators identified from network analysis. The inhibition of viability was determined by live/dead staining of 20 h-old static biofilms of mutant, wild type and *cysR* complementation strains under carolacton treatment and is expressed as inhibition of the green/red fluorescence ratio. The bars show the mean of three independent biological replicates. **(B)** The effect of carolacton treatment on the number of colony forming units (cfu’s) of biofilms of the *S. mutans* UA159 wildtype and *cysR* gene deletion mutant was also investigated. The Cfu experiment was repeated in two biological replicates. The sequences of primers used for generating the deletion mutants are given in Additional file 9.

The susceptibility to carolacton treatment of 20 h-old static biofilms of the corresponding key regulator gene deletion mutants was tested using Live/Dead viability staining. As shown in Figure 7A, carolacton only marginally reduced the viability (~5-10% inhibition) of the biofilms of the *cysR* gene deletion strain, while complementation of *cysR* in trans fully restored the carolacton sensitive phenotype of the wildtype (approximately 45-55% inhibition of viability). It was previously shown that carolacton exclusively damages growing biofilms [5]. The observed strong loss in sensitivity of the *cysR* gene deletion strain to carolacton was not biased due to poor or significantly slower growing mutant biofilms since the final growth yield and doubling time of the *cysR* gene deletion mutant was only slightly reduced in comparison to the wildtype (data not shown). Strikingly, the *spxA* gene deletion strain displayed impaired ability to grow under acidic conditions [92], a condition known to be essential for the carolacton induced membrane damage in *S. mutans*[5]. However, all the tested strains with the *fabT* or *cysR* deletion showed similar susceptibilities to carolacton relative to the wildtype (Figure 7A). For the *fabT* deletion mutant though, no final conclusion regarding its sensitivity to carolacton treatment could be drawn since the mutant strain grew very poorly under the tested conditions and formed very thin biofilms. Since growth is a prerequisite for the membrane damage caused by carolacton treatment [5], the results of the Live/Dead staining for the *fabT* mutant should be interpreted with caution. However, the membrane integrity of the *fabT* deletion mutant was highly compromised (data not shown), as determined by live/dead staining. The importance of *fabT* for maintaining membrane integrity in *S. mutans* biofilms might also be indicative of its involvement in the membrane damage caused by carolacton treatment. Furthermore, *fabT* was identified as a top structural hub of the inferred co-expression network, indicating its essential physiological role, and the deletion of which is therefore expected to have a high chance to cause poor growth.

### The role of *cysR* in the response of *S. mutans* biofilms to carolacton

From our initial susceptibility studies of mutant biofilms under carolacton treatment, *cysR* could be identified as being essential for the sensitivity of growing *S. mutans* biofilms to carolacton treatment. The almost complete loss of sensitivity of the *cysR* mutant to carolacton treatment was independently confirmed by the determination of colony forming units of carolacton treated and untreated biofilms of the wild type and *cysR* gene deletion strains. While the wildtype showed a reduction of CFUs of approximately 75%, the carolacton treated and untreated mutant cells showed almost no difference in the amount of colony forming units (Figure 7B). Thus the experiment clearly confirms the essential role of *cysR* in the response of *S. mutans* biofilms to carolacton treatment, as predicted from the TRRN. The genome of *S. mutans* encodes 4 LysR-type transcriptional regulators, of which 3 (CysR, MetR, HomR) are phylogenetically linked and control the supply of sulfur amino acids [81, 93]. CysR is known to modulate the expression of genes involved in cysteine metabolism in *S. mutans*[81]. Here, we provide data showing that apart from its role in cysteine metabolism, CysR is essential for the response of *S. mutans* biofilms to the biofilm inhibitor carolacton. This implies a potential role of CysR in cell wall maintenance, as this trait was shown to be impaired in carolacton treated biofilm cells and cell wall changes are most likely responsible for cell death caused under acidic conditions [5].

The inferred transcriptional regulatory targets of CysR, as identified after the integration of contextual coexpression correlation and binding motif information, indeed comprise genes involved in maintaining cell wall homeostasis. Genes SMU_984 and SMU_609 encode two autolysins which were not characterised so far. Interestingly, both genes were immediately (5 min after treatment) and constantly upregulated after carolacton treatment and belong to the strongest regulated genes of our time-resolved transcriptome study. SMU_609 encodes a 40 K cell wall protein which was shown to exhibit murein hydrolase activity using a peptidoglycan zymogram assay and is likely linked to the cell surface via the sortase system [78, 94]. As cell wall synthesis during growth depends on a balanced interplay between build-up and breakdown of the cell-wall, enhanced autolysin activity might promote cell lysis [95] and thus explain the influence of CysR on cell death. However, deletion of SMU_609 and SMU_984 did not cause a carolacton insensitive phenotype (data not shown). Another CysR regulated gene, *rgpG* (SMU_246), encodes a putative UDP-N-acetylglucosamine undecaprenyl-phosphate GlcNac 1-phosphate transferase. This enzyme catalyses the first membrane localized step of the biosynthesis of various polymers of the bacterial cell wall. The other potential target genes include the gene cluster SMU_1074-SMU_1077 which encode putative metabolic enzymes belonging to the pathways related to cysteine metabolism and is concurrent with the known role of *cysR* in cysteine metabolism [81].

It should also be noted that *cysR* and its paralog *cpsY* are located in close vicinity to the genes of the de novo pyrimidine biosynthesis pathway on the genome of *S. mutans*. The pyrimidine biosynthesis genes were shown to be the strongest upregulated genes 5 min post carolacton treatment. The functionality of pyrimidine metabolism, especially of the *pyr* gene clusters upregulated specifically under carolacton treatment, could possibly be linked to the availability and synthesis of cell wall precursors. In prokaryotes, genes encoding products involved in the same or related biological functions are often located in close neighbourhoods on the genome [96–98].

Moreover, for the *S. iniae cysR* ortholog *cpsY*, it was shown that this LysR type transcriptional regulator (LTTR) induced cell wall changes essential for the intracellular survival of this invasive pathogen in neutrophils [99]. Interestingly, in the study of Allen *et al.*[99], it was determined that deletion of *cpsY* alters the cell surface charge, muropeptide composition and susceptibility to lysozyme treatment. This is fully in accordance with the current understanding of the mode of action of carolacton [5]. However further experiments are necessary to verify the targets of CysR experimentally (e.g. EMSA experiments with purified CysR protein) and to fully address the role of *cysR* in cell wall metabolism e.g. by knocking out the CysR regulated autolysins. Altogether, our data strongly suggest a so far unidentified role of the CysR transcriptional regulator in cell wall homeostasis.

A highly interesting aspect of the network prediction is the finding that CysR potentially regulates another transcriptional regulator (Rgg). As the carolacton specific regulatory response network contains albeit only two levels of hierarchy, CysR can thus be considered as a potential global regulator. Rgg transcriptional regulators associated with small hydrophobic peptides (SHP) were identified to represent a part of a novel quorum sensing mechanism in Streptococci [82, 100]. Rgg proteins have been identified and conserved in nearly all streptococci [101]. This mechanism works independently of a TCS, but senses the signalling molecule inside the cell after its internalisation via an oligopeptide permease [82]. But the Rgg knockout strain showed comparable susceptibility to carolacton treatment as the wildtype strain (Figure 7A) thus excluding the possibility of Rgg being a critical regulator mediating the observed effects of carolacton.

With respect to *cysR* however, its deletion mutant displayed a loss of sensitivity to carolacton as a result of a reduction in the inhibition of viability caused by carolacton. Nevertheless, CysR might not represent the primary target of carolacton as the treated biofilm cells of the *cysR* gene deletion mutant still exhibit an inhibition of about 10%. Moreover, phenotypic differences between carolacton treated and untreated *cysR* mutant biofilm cells were observed, indicating that carolacton can still bind to its target(s) and induce changes in the cellular morphology. Nevertheless, the lethal effects of carolacton treatment apparently rely to a large extent on the presence of an intact copy of the *cysR* gene. Another interesting question is the link of CysR to the PknB controlled regulon, as *pknB* was also shown to be essential for carolacton mediated cell death [5]. With PknB being a global regulator of cell division [102] and virulence attributes [103], the regulons of PknB and CysR might converge at the level of the modulated autolysins. The CysR coregulated genes SMU_984 and SMU_609 were 2 of the 3 strongest modulated genes in the transcriptome analysis of an exponentially growing *pknB* deletion strain [103]. However, as the deletion mutants of SMU_609 and SMU_984 are still susceptible to carolacton treatment, this potential overlap of the regulatory systems does not provide an explanation why both the *pknB* and *cysR* deletion mutant biofilms exhibited almost no sensitivity to carolacton treatment.

Since LTTRs show a high degree of sequence conservation, control important metabolic pathways of sulfur containing amino acids, and since sulfur is essential for the active sites of many enzymes, the transcriptional regulator CysR might also be an attractive drug target. Co-factor binding is required for its transcriptional activation, and the likely cofactor could be O-Acetylserine (OAS) [81]. Thus, small molecule inhibitors could potentially be designed to compete with the co-effector molecule for the binding site on this LTTR and to finally block its biological function. To conclude, we have shown here strong evidence that the role of CysR may be much more diverse and important than previously expected. Traits essential for survival like cell wall metabolism and sulfur amino acid supply are controlled by this LTTR. Thus CysR might represent an attractive novel streptococcal drug target.

### Linkage between the PknB and CysR regulons

Deletion of *cysR* almost completely prevents cell death in carolacton treated biofilms, a finding previously reported for the gene deletion strain of the Serine Threonine Protein Kinase (STPK) encoding gene *pknB* as well [5]. This instantly raises the question whether the regulons of these two proteins overlap or if PknB is located upstream of CysR in the regulatory cascade and thus controls the latter’s activity. Both regulators putatively modulate the expression of the two carolacton responsive autolysins SMU_984 and SMU_609, which might explain the shared insensitive phenotype to carolacton of their mutant strains.

However, in this study, we focus only on the transcriptional network analysis, while PknB modulates its target genes via protein phosphorylation at the post-transcriptional level [9, 103, 104]. Thus the question whether and on which level the modulated networks of PknB and CysR converge and if they represent the essential pathway for the carolacton mediated cell death cannot be fully answered from the transcriptional regulatory network analysis. To quantitatively understand the effects of carolacton on cell metabolism and to determine the missing layers in the regulatory cascades of CysR and PknB, it is necessary to consider the regulatory effects of small non-coding RNAs [105] and other post-transcriptional and post-translational modifications [106, 107] including modulation by other signalling pathways [108]. Transcriptional network analysis is generally lacking in information about these important regulatory mechanisms which have been shown to be essential for cellular metabolism and homeostasis [105, 106, 109]. Nevertheless, we have demonstrated here the high merit of transcriptional network prediction and analysis to identify a key regulator mediating cell death in *S. mutans* biofilms in response to the biofilm inhibitor carolacton.

## Conclusion

We have used a combination of transcriptional network prediction and experimental verification to analyse the response of *S. mutans* biofilms upon treatment with the biofilm inhibitor carolacton. According to our knowledge, this is the first study inferring a genome-wide transcriptional regulatory response network for *S. mutans* using heterogeneous data including a time-series transcriptomic dataset and transcription factor binding site information*.* Based on the inferences from the regulatory network, we experimentally verified important predicted transcriptional regulatory interactions between the cell envelope stress modulating TCS response regulator MbrC and the operon harbouring the *murMN* genes encoding cell wall metabolism associated enzymes. CysR, a regulator known to modulate cysteine metabolism, was predicted and experimentally verified to be an essential key regulator for the mode of action of the biofilm inhibitor carolacton. Our data strongly suggest a role of *cysR* in cell wall metabolism, cell division and cell surface biogenesis, apart from its already known role in cysteine metabolism and sulfur supply in *S. mutans,* thus highlighting its potential as an attractive novel streptococcal drug target. We here also demonstrate the high predictive power of the network construction and analysis strategy used in this work.

## Methods

### Transcriptomics

Static biofilm cultures of *S. mutans* UA159 were grown in routine 24 well plates (Greiner Bio One, Frickenhausen, Germany) using 800 μl Todd Hewitt Broth (Becton Dickinson, Heidelberg, Germany) supplemented with 0.5% sucrose (Sigma, Taufkirchen, Germany) per well as media. Overnight cultures of planktonic cells in Todd Hewitt Broth were diluted in the biofilm medium to a starting OD of 0.05 and biofilms were grown for 3 hours under anaerobic conditions (80% N_2_, 10% CO_2_, 10% H_2_) in a chamber (Don Whitley Scientific, Shipley, England). Thereafter, the supernatants were completely removed and exchanged with fresh medium (control samples) or either fresh medium supplemented with 2.5 μg/ml carolacton (treated samples). For the control, an equivalent volume of methanol was added. Samples were taken after 0, 5, 20, 40, 60, 80, 100, 120, 160, 200, 240 and 300 minutes by removing the supernatant and adding 200 μl fresh medium and 400 μl of RNA protect (Qiagen, Hilden Germany). Biofilms representing one condition were scraped from at least 3 individual wells and pooled together. Cells were pelleted by centrifugation, washed with Molecular biology grade water (Sigma, Taufkirchen, Germany) and lysed with a combination of Lysozyme/mutanolysin treatment and mechanical disruption of cells with glass beads [5]. Total RNA was extracted using the RNeasy Kit (Qiagen, Hilden Germany) as recommended by the manufacturer. The overall experiment was repeated once.

2 μg of total RNA from each sample was labeled with Cy3 and Cy5-ULS (Kreatech, Netherlands) according to the protocol of the company. Fragmentation of RNA and further processing was performed according to the Agilent hybridization kit procedure. Samples were hybridized in a dye swap design on 8x15K *S. mutans* custom arrays (Agilent, Böblingen, Germany) for 14 h at 65°C as previously described [5].

The array design is described in detail in [5]. Slides were washed and scanned as recommended by Agilent. For feature extraction, the Agilent extraction software (V. 10.7) was used. The further data analysis was performed using the R package LIMMA. For dye specific within-array normalization, the LOWESS algorithm was applied. Quantile normalization was used for between array-normalization. Genes corresponding to rows with blanks were omitted. From the log transformed and background corrected median signal intensities, the fold change between treated and untreated samples was calculated for every gene at each time point. The log2 (treated/untreated) expression ratios were then subjected to a within-gene normalization. Genes with a log2-fold ratio greater than 0.8 were regarded as genes corresponding to transcripts with altered abundances (GTAAs).

### Functional context enrichment analysis

Enrichment analysis was performed separately for the up- and downregulated GTAAs (at every measured time point) with respect to each of the known biological functional classes and KEGG metabolic pathways in order to gain an overview of the various functional categories affected by carolacton treatment. 15 main and 97 sub functional classes of *S. mutans* UA159 were compiled using the gene annotation information furnished in the Oralgen database (version 2011 and now re-named as The Bioinformatic Resource for Oral Pathogens). The gene membership information of *S. mutans* UA159 corresponding to 84 different pathways was retrieved from the KEGG database (version 2011). The hypergeometric test with Benjamini-Hochberg correction was used to quantify the significance of the overlap between the GTAAs and the functional categories. A corresponding significance score was also calculated as a negative logarithmic function of the corrected P value. The significance score is a user friendly measure of the relevance of the overlap between functional categories and the GTAAs. The higher the significance score, the more relevant is the overlap. Categories with significance score greater than 0 represented significant enrichment.

### Inferring gene-gene correlation relationships from transcriptomic data using the Trend Correlation (TC) method

The TC method [32] allows the inference of gene-to-gene time-lagged positive or negative “associations” or pairs of genes based on extracting the main features of the change trend and the correlation of gene expression changes between consecutive time points [32]. Time lagged co-expression relationships are those in which the expression patterns of two genes are better correlated when a time shift is introduced [32]. In this work, we adapted the TC method by calculating the correlation coefficient from the change rates rather than change levels between consecutive time points since the time interval of the measurements was not constant (personal communication, Feng He). Potential co-expression relationships were checked for every possible regulator-gene pair given the minimum number of expression measurements in the chosen time window was four. The genetic response network consisted of optimal expression relationships which started either at 0, 5 or 20 min post carolacton treatment in order to capture the correlations mediating the initial response to carolacton. The significance of correlation of a gene-pair for every time-window is measured by the co-expression P-value by procedures described previously in the TC method by He et al. [32]. The individual P-values of correlation for a gene pair within every considered time-window are compared among each other. Optimal correlation for a gene pair refers to the time window within which the correlation between the genes is most significant statistically. Parameters such as the correlation co-efficient, the trend score, and the P-value were calculated as described previously [32] to characterize the degree and randomness of correlation between the temporal expression patterns of genes. The association between two genes was considered to be statistically significant if the corresponding P-value of their co-expression was determined to be lower than the optimal P-value cutoff calculated using standard performance metrics as explained below.

### Performance metrics for the determination of the optimal P-value threshold

The optimality of the P-value threshold was determined based on the F-score (Eq. 1) which in turn is a function of the equilibrium between the Recall (Eq. 2) and Precision (Eq. 3) functions which are defined below.

-  F-score: this is predictive of the balancing property or equilibrium between the Precision and Recall parameters. The F-score usually reaches a maximum peak at the P-value optimum.1
-  Recall (also termed as True Positive Rate or Sensitivity): defined as the fraction of true “associations” which are inferred as significant by the TC method2
-  Precision (also termed as Positive Predictive Value): defined as the fraction of inferred significant “associations” which are true3

Gene pairs within the same operon because of their virtue of coherent co-expression were used as the “gold standard” or true positives. False positives constitute the inferred significant “associations” between genes from different operons whereas false negatives represent the intraoperonic gene pairs which were considered as insignificant and which were seemingly missed out at the particular P-value threshold. The operon information was predicted by Pathway Tools [110]. Differential edges [111, 112] in response to carolacton treatment were pooled together to form the final co-expression network.

### Construction of Position Specific Scoring Matrices (PSSMs) representing transcription factor binding motifs

Experimentally verified DNA binding sites of *S. mutans* transcription factors were retrieved from literature when available. The comparitive genomics based approach using the concept of regulogic extrapolation as explained in [33] was carried out to retrieve orthologous regulatory relationships in *S. mutans* from experimentally verified relationships in *B. subtilis*. Validated binding sites of *B. subtilis* transcription factors were retrieved from the DBTBS database [113]. OrthoMCL [114], which is a genome-scale algorithm for grouping matching protein sequences, was used to identify orthologous genes shared between *S. mutans* UA159 and other gram positive bacterial species including some representative species from the Streptococcus genus. The OrthoMCL results were verified by manually searching for the presence of commonly occurring domains in the protein sequences of the orthologous pairs of transcription factors. To increase the reliability of a particular predicted regulog in *S. mutans*, a check was performed to ascertain if the regulog was conserved in at least some species within the Streptococcus genus. Consensus motifs constructed from too few binding sites have a low predictive value [37]. Hence the available binding sites were enriched with corresponding putative sites from regulogs identified from different species within the Streptococcus genus. In addition to the PSSMs constructed from manually identified regulogs, PSSMs were also compiled from already assembled regulogic sites retrieved from the RegPrecise database [36] and from phylogenetic footprinting studies. Each resulting set of binding sites was submitted to *consensus*, which is a tool for mining out conserved motifs. The tab-formatted PSSMs were then converted into TRANSFAC formatted PSSMs which enable an user-friendly organization of pattern matching results. Additional file 3 lists the transcription factors with their corresponding binding site information.

### Prediction of putative binding sites by Pattern Matching

Since the predictive capacity of each PSSM (due to its varying information content) differs from case to case, a generic or common P-value could not be used to screen out “putative-target hits” (i.e. genes or operons harboring binding sites predicted by the corresponding PSSMs). The RSAT tool termed *matrix-quality*[37] was used to objectively determine the optimal P-value for every PSSM. The TRANSFAC formatted PSSMs were then employed to search for putative sites using the tool *matrix-scan* (http://www.rsat.eu/) [115, 116] with the determined optimal P-values set as the cut-offs. Putative sites were searched for in the upstream regions (1000 bps upstream of the start codon with no overlap with the coding regions of the preceding ORF) of all genes in the *S. mutans* UA159 genome. A markov order of 0 and a background model comprising *S. mutans* specific upstream regions (no overlap with ORF coding regions) was used during the pattern matching scan. The resulting sites with a P-value smaller than the determined optimal P-value were considered as significant. In cases of PSSMs for which the optimal P-values could not be determined due to the inherent low complexity of the motifs [37], a whole genome string-based site identification was performed using the non-matrix based tool genome-scale *dna pattern* (http://www.rsat.eu/) [115] with an allowance for a maximum of 2 mismatches. Both the forward and reverse strands were subject to the pattern matching procedure.

### Analysis of sub-networks

Even if the expression profiles of not all the genes in the same operon are well correlated with the expression of a certain regulator gene, we assume that all the genes in the same operon will be regulated by the regulator provided that the corresponding binding sites are found in the upstream regulatory regions of the operon. This is what we refer to as operon-oriented adjustment.

To obtain the TRRN, the co-expression network was superimposed onto the regulator-target gene binding site map. The genes in each co-regulated TRRN group or subnetwork after operon-oriented adjustment were analyzed to detect any significant over-representation of categories such as biological functional classes, KEGG metabolic pathways and gene ontology terms. The hypergeometric distribution was employed to determine over-represented categories with the network analysis tool *compare-classes* (http://www.rsat.eu/). Self and reciprocal comparisons were avoided. Enrichment events with significance scores > =0 were considered as statistically significant. Gene ontology information was retrieved from the Pathosystems Resource Integration Center (PATRIC) [117]. All the network visualizations were performed using Cytoscape [118]. Network motifs were analyzed using the tool MFINDER [119]. The significance of the discovered motifs in the real network was determined in comparison to their occurrences after 10000 randomizations (at a P-value < =0.01). Heat map representation of metabolic pathways was generated using the Mayday visualization tool version 2.12.

### Heterologous expression and purification of response regulator proteins

Coding sequences corresponding to the *S. mutans* response regulators MbrC (SMU_1008) and VicR (SMU_1517c) were PCR-amplified with Phusion-Polymerase (NEB) using primer pairs MbrCFor/MbrCRev and VicRFor/VicRRev respectively (see Additional file 9). Homologous flanks to the vector sequence of pET28c were implemented at the 5’ ends of the primer. The purified DNA was subsequently cloned in expression vector pET28c (Novagen) cut with NcoI/XhoI (Fermentas) using the CloneEZKit (Genescript). Resulting plasmids bearing the coding sequence for a N-terminal His-Tag were verified by sequencing and transformed in BL21Star. Cells were grown in 4 l shaking flaks, induced with 1 mM IPTG at an OD_600_ of 0.5 and harvested 4 hours after induction. The resulting cell pellet was re-suspended in lysis buffer (50 mM Tris, 150 mM NaCl, pH 7.4) supplemented with 4 mg/ml lysozyme (Sigma) and frozen at −20°C. After de-freezing the pellet, 100 μg DNAse I (Roche) and 5 mM MgSO_4_ (Sigma, final concentration) were added and 5 cycles of sonification for 60 seconds were applied to lyse cells (duty cycle 0.5 Sec, pause 1 sec, amplitude 60%). Finally 0.1% NP40 (Sigma, final concentration) and 10 mM imidazol (Sigma, final concentration) were added and cell debris was removed by centrifugation and filtration (0.45 μm). His-tagged proteins were purified on a HIS-Talon (Clontech) column using a Duo Flow FPLC (Biorad) at a flow rate of 0.5 ml/h. Resulting purified proteins were desalted using PD10 columns (Amersham), eluted in 1xPBS buffer and measured using a spectrophotometer (Nanodrop ND1000, Peqlab) to determine the protein concentration. The response regulator was over 99% pure as confirmed by SDS-PAGE (data not shown). Promoter regions containing the potential binding sites of MbrC were PCR amplified using Taq-polymerase. The primers used are listed in Additional file 9. PCR-products were purified using the PCR-Purification Kit (Qiagen) and used as targets for EMSA.

### Deletion of potential binding sites

Potential binding sites of the response regulator MbrC were deleted using a PCR-driven overlap extension method [120]. Motifs in the promoter regions of SMU_610, SMU_718, and SMU_1006 were deleted by two initial PCRs generating overlapping DNA sequences. Taq polymerase (Qiagen) was used for the amplification of regions flanking the potential binding sites. PCR-products were purified from an agarose gel using the Gel Extraction Kit (Qiagen) and used as template for a third PCR with Phusion polymerase using primers (Additional file 9) spanning the whole region. Purified PCR-products were cloned blunt-end in the EcoRV restriction site of vector pGEM5Zf(+) (Promega). Resulting plasmids were verified by sequencing and used as template for a PCR to finally generate the DNA-targets for EMSA.

### EMSA procedure

The response regulator (MbrC and VicR) proteins were activated by acetylphosphate (Sigma). An aliqot of the protein was incubated for 2 h at RT (room temperature) in reaction buffer (25 mM acetylphosphate, 50 mM Tris–HCl, 50 mM KCl, 10 mM MgCl_2_, 4 mM dithiothreitol) as described previously. Excess of acetylphosphate was removed by filtration and the protein was serially diluted in binding buffer (10 mM Tris, 1 mM EDTA, 100 mM KCl, 100 μM DTT, 5% vol/vol glycerol, 10 μg/ml BSA, pH 7.5). 0.5 pmol of target and competitor DNA was added to each reaction and incubated for 1 h at RT. The unrelated response regulator VicR which has a similar MW and pI as the response regulator MbrC under study was used as a negative control to rule out unspecific DNA binding. Four μl of the reaction mixture was applied on a 5% acrylamide gel run in Tris-borate-EDTA (TBE) buffer at pH 7.4. Gels were stained using SybrGold and visualized in a transilluminator (Alpha DigiDoc, Biorad) at 254 nm.

### Construction of gene deletion mutants and a *cysR* complementation strain

Upstream and downstream flanking regions of *cysR* were PCR-amplified using primers CysR P1/2 and CysR P3/4 (see Additional file 9 for the primers used to generate deletion mutants) and genomic DNA of *S. mutans* UA159 as template. The erythromycin resistance cassette was amplified from genomic DNA of a previously constructed mutant [5]. Restriction sites of AscI and FseI were introduced via the 5’-termini of the primers. After restriction digestion of purified PCR-products with the appropriate restriction enzymes, the up and downstream flanks were ligated to the ERM cassette using T4 DNA Ligase and directly transformed in the *S. mutans* UA159 WT strain according to the procedure of Li et al. [121]. Gene deletion strains were selected on THBY agar plates containing 10 μg/ml erythromycin. Genomic DNA of isolated clones was analysed with PCR and primers P1 and P4 spanning the entire gene deletion construct. Sequencing was used to further confirm the correct insertion of the gene deletion construct via double homologous recombination. For the generation of the four other gene deletion strains (*glnR*, *fabT*, *rgg* and *spxA*), a similar procedure was carried out.

RNA was isolated from the exponentially growing *cysR* gene deletion strain and was verified for the absence of *cysR* mRNA with RT-PCR using the 3 primer pairs Q-CYSR1-F/R, Q-CYSR2-F/R, and Q-CYSR3-F/R. Primers specific for the ComX (ComX F/R) and ComE (ComE F/R) encoding genes were used as positive controls for the RT-PCR reaction. Reactions in which the reverse transcriptase was replaced with water functioned as negative controls. For the complementation of the *cysR* gene deletion strain, the entire *cysR* gene including its promoter region were PCR-amplified with primers C-CYSR1-F and C-CYSR1-R and cloned blunt end via the SmaI restriction site in the replicative plasmid pDL278 [122]. Resulting plasmids were verified by sequencing and transformed in the *cysR* gene deletion strain as described elsewhere [121].

### Viability measurements of the carolacton treated *cysR* deletion strain using Live/Dead viability staining and by Cfu determination

Viability measurements using live/dead viability staining of carolacton treated and untreated biofilms of the *S. mutans* UA159 wildtype, gene deletion and complementation strains were essentially performed as previously described [5]. Biofilms of *S. mutans* UA159 WT were used as positive control. The experiment was repeated in two biological replicates. For the cfu determination, biofilms representing one condition were harvested and pooled from 5 individual wells. Chains and biofilm clumps were dispersed and destroyed using mild ultrasonification conditions as described before [123] and plated on THBY-agar plates (containing 10 μg/ml erythromycin for the mutant) in 3 technical replicates. Live dead staining before and after sonification verified that the sonification had no significant influence on cell viability. The overall experiment was repeated in 2 biological replicates.

## Electronic supplementary material

Additional file 1: **Functional enrichment analysis (with respect to biological functional classes and KEGG metabolic pathways) of the set of up- and downregulated GTAAs upon carolacton treatment at each time point.** The biological functional classes derived from the Oralgen database (version 2011 and now re-named as The Bioinformatic Resource for Oral Pathogens) were classified as main or sub-functional classes depending on their annotation hierarchies whereas the pathway information was derived from the KEGG database (version 2011). Functional classes that are most significantly enriched (significance score > =1) are marked in dark green and those with decreasing significance with diminishing shades of green under the threshold limitation of significance score > =0. The hypergeometric distribution with Benjamini-Hochberg correction was used to determine the significance of over-representation. NA indicates the absence of GTAAs at the given log2-fold ratio cutoff or the absence of statistically significant over-representation (significance score < 0) of functional categories. (XLS 114 KB)

Additional file 2: **The** ***S. mutans*** **genetic co-expression network in response to carolacton treatment as inferred using the Trend Correlation method.** 8284 gene pairs were inferred as statistically significant by using the standard performance metrics and procedures as described in the Methods section. The P-value indicates the randomness of the inferred gene-gene co-expression relationships. Positive co-expression relationships between genes are denoted by 1 and negative inverted relationships by 0 under the ‘Relationship’ column. (XLSX 610 KB)

Additional file 3: **The list of** ***S. mutans*** **UA159 transcription factors and their binding motif information compiled in this study.** TF: Transcription factor. BS: *Bacillus subtilis*. AAA (SMART database accession number SM00382): Domain found in ATPases associated with a variety of cellular processes; Bac_DnaA_C (SMU00760): Domain representing the C-terminal regions of bacterial DnaA proteins; PDZ (SM00228): Domain representing distinct regions found in diverse signalling proteins; REC (SM00448): Type of response regulator domain; HTH_LUXR (SM00421): DNA-bending helix-turn-helix domain present in transcriptional regulators of the LuxR/FixJ family of response regulators. HTH_MERR (SM00422): Domain found in transcriptional regulators which bind DNA via a helix-turn-helix structure and which mediate mercury-dependent induction of the mercury-resistance operon. CoA_binding (SM00881): Domain found in a number of proteins including succinyl CoA synthases, malate and ATP-citrate ligases. HTH_ARSR (SM00418): DNA-binding, winged helix-turn-helix (wHTH) domain present in transcription regulators of the arsR/smtB family and involved in stress-response to heavy metal ions; Trans_Reg_C (SM00862): Domain found in the C-terminal regions of response regulator proteins. (DOCX 61 KB)

Additional file 4: **The list of regulator-target gene relationships based on the prediction of binding sites using Pattern Matching.** Please refer to the Methods section for more information on the Pattern Matching procedure. (XLSX 211 KB)

Additional file 5: **Data representing the transcriptional regulatory response network (TRRN) of** ***S. mutans*** **biofilms upon treatment with the biofilm inhibitor carolacton.** The table displays the co-expression characteristics of significant correlations as well as the information corresponding to the predicted transcription factor binding sites on the upstream regulatory regions of the target genes or their corresponding operons. NA*: motifs predicted using the pattern matching tool *dna pattern*. Positive relationship between the genes is denoted by 1 and negative inverted relationships by 0 under the Relationship column. (XLSX 43 KB)

Additional file 6: **The different types of local network motifs identified in the** ***S. mutans*** **TRRN upon carolacton treatment.** The motifs were identified and their corresponding statistical scores determined using the MFINDER tool [47, 119] after 10000 randomizations. *SD denotes the standard deviation of the number of occurrences of the motif in randomized networks. ^The “Z score” is a measure of the statistical significance of the motifs and is determined as (M_real_ - M_rand_)/SD. Motifs with P-values < = 0.01 were considered as significant. (DOCX 46 KB)

Additional file 7: **List of enriched categories in the co-regulated gene groups or subnetworks of the** ***S. mutans*** **TRRN upon carolacton treatment.** Over-represented categories include KEGG metabolic pathways (KEGG MP), Biological functional classes (BFC) and gene ontology terms (GO). The details pertaining to the calculation of the significance scores are provided under the Methods section. (DOCX 58 KB)

Additional file 8: **The normalized expression profiles of the genes co-regulated by the global transcription factors CcpA and CodY.** For the CodY co-regulated gene group, only the expression profiles of genes with positive relationships are shown. (PNG 755 KB)

Additional file 9: **Primers used in the study.** (DOCX 36 KB)

## Abbreviations

TRRN: Transcriptional regulatory response network
EMSA: Electro mobility shift assay
TCS: Two component system
RR: Response regulator
*gtf*: glucosyltransferase
*gbpB*: glucan binding protein B
TSNI: Time series network identification
GTAA: Genes corresponding to transcripts with altered abundances
KEGG: Kyoto encyclopedia of genes and genomes
TC Method: Trend Correlation method
ORF: Open reading frame
PATRIC: Pathosystems resource integratrion center
WT: Wild type
RT-PCR: Reverse transcriptase polymerase chain reaction
SDS-PAGE: Sodium Do-decyl Sulfate Poly-Acrylamide Gel Electrophoresis
PSSM: Position specific scoring matrix
RSAT: Regulatory sequence analysis tools
GTP: Guanosine tri phosphate
SIM: Single input module
MIM: Multiple input module
FFL: Feed forward loop
MOFFL: Multi-output Ffl
UDP: Uridine di phosphate
UTP: Uridine tri phosphate
UMP: Uridine mono phosphate
UDP-N-AG: Uridine Di-Phosphate N-Acetyl Glucosamine
ATR: Acid tolerance response
CFU: Colony forming units
LTTR: LysR type transcriptional regulators
XIP: sigX inducing peptide
OAS: O-acetylserine.
