# Erratum to: Construction and verification of the transcriptional regulatory response network of *Streptococcus mutans* upon treatment with the biofilm inhibitor carolacton

**DOI:** 10.1186/1471-2164-15-739

**Published:** 2014-08-29

**Authors:** Padhmanand Sudhakar, Michael Reck, Wei Wang, Feng Q He, Irene Wagner-Döbler, An-Ping Zeng

**Affiliations:** Institute of Bioprocess and Biosystems Engineering, Hamburg University of Technology, 21073 Hamburg, Germany; Research Group Microbial Communication, Helmholtz Center for Infection Research, Inhoffenstrasse 7, 38124 Braunschweig, Germany; Luxembourg Centre for Systems Biomedicine, 7, Avenue des Hauts Fourneaux, L-4362 Belval, Luxembourg

## Correction

After publication of the original article [1] it came to the publishers attention that one name had been inadvertently misspelt. The correct authors’ list should read as indicated above. We apologize for any inconvenience this has caused.
