# Supplementary material for: Construction and verification of the transcriptional regulatory response network of Streptococcus mutans upon treatment with the biofilm inhibitor carolacton
Source: BMC Genomics. 2014 May 12;15:362. doi: 10.1186/1471-2164-15-362 (PMC4048456; doi:10.1186/1471-2164-15-362)
Supplement: Supplementary file 3 — Additional file 3: The list of S. mutans UA159 transcription factors and their binding motif information compiled in this study. TF: Transcription factor. BS: Bacillus subtilis. AAA (SMART database accession number SM00382): Domain found in ATPases associated with a variety of cellular processes; Bac_DnaA_C (SMU00760): Domain representing the C-terminal regions of bacterial DnaA proteins; PDZ (SM00228): Domain representing distinct regions found in diverse signalling proteins; REC (SM00448): Type of response regulator domain; HTH_LUXR (SM00421): DNA-bending helix-turn-helix domain present in transcriptional regulators of the LuxR/FixJ family of response regulators. HTH_MERR (SM00422): Domain found in transcriptional regulators which bind DNA via a helix-turn-helix structure and which mediate mercury-dependent induction of the mercury-resistance operon. CoA_binding (SM00881): Domain found in a number of proteins including succinyl CoA synthases, malate and ATP-citrate ligases. HTH_ARSR (SM00418): DNA-binding, winged helix-turn-helix (wHTH) domain present in transcription regulators of the arsR/smtB family and involved in stress-response to heavy metal ions; Trans_Reg_C (SM00862): Domain found in the C-terminal regions of response regulator proteins. (DOCX 61 KB) [file 12864_2013_6097_MOESM3_ESM.docx]

DNA binding motifs for 44 transcription factors were mined out based either on the experimentally verified binding sites in S.mutans or using comparative genomics approaches employing the concept of regulogs [37]. *When binding sites were extrapolated regulogically from B.subtilis, the information such as sequence conservation, E-value, and commonly present domains (according to SMART database) between the B.subtilis transcription factor and its ortholog in S. mutans UA159 are shown. Sites from the regulogs in other species within the Streptococcus genus were used to complement the regulogic sites in S.mutans so as to improve the predictive power and information content of the resulting PSSMs. PSSMs from the REGPRECISE database [40] were derived from already enriched sets of regulogic sites. °indicates spaced motifs for which motif width is not a suitable indicative parameter. ^The optimal P-value and optimal weight score for PSSMs was determined from the matrix-quality tool [41] analysis and subsequently used for pattern matching. For PSSMs for which the optimal P-values could not be determined as a result of the low complexity of the motifs, a string-search based tool called genome-scale dna pattern was used to determine putative sites. These transcription factors are denoted by ‘DP’ under the optimal P-value and optimal-weight score columns.

| **The list of S. mutans UA159 transcription factors and their binding motif information compiled in this study** | | | | | | | | | |
| --- | --- | --- | --- | --- | --- | --- | --- | --- | --- |
| **TF name** | **Locus tag of S. mutans UA 159 TF** | **TF description** | **Source of motifs*** | **Motif width** | **IUPAC TF binding motif consensus** | **Total information content of PSSM** | **Information content per column** | **Optimal P-value^** | **Optimal weight score^** |
| AdcR | SMU_1995c | Putative transcriptional regulator of zinc homeostasis | Regprecise | 16 | amtTaACYRGTtaAkt | 12.0655 | 0.75409375 | 1.80E-05 | 8.9 |
| ArgR | SMU_2097 | Transcriptional regulator of arginine biosynthesis and degradation | Regprecise | 16 | ttKtatawwwAtaMaa | 7.78467 | 0.486541875 | 2.40E-05 | 9 |
| BirA | SMU_1578 | Biotin biosynthesis regulator | Regprecise | NA° | ATaGTTAAC-(16)-GTTAACtAa | -- | -- | DP | DP |
| CcpA | SMU_1591 | Catabolite control protein A. | Regprecise | 16 | wtGaaArCGyTttCaw | 9.59376 | 0.59961 | 2.60E-04 | 8 |
| CiaR | SMU_1129 | Two component response regulator | Regprecise | NA° | TTTAAG-(5)-aTTAAG | -- | -- | DP | DP |
| CodY | SMU_1824c | Transcriptional repressor of amino acid metabolism | Regprecise | 15 | aattTTCwGAawaTT | 10.6889 | 0.712593333 | 4.93E-04 | 5.1 |
| ComE | SMU_1917 | Response regulator of the competence regulon | Ref [72] | 11 | rGGaTTkrCCa | 9.49081 | 0.862800909 | 9.96E-05 | 5.8 |
| CopY | SMU_424 | Negative copper transcriptional regulator | Regprecise | 20 | tmRtytACAaatGTArayww | 12.9104 | 0.64552 | 4.08E-06 | 11 |
| CtsR | SMU_2030 | Putative transcriptional regulator of heat shock response | Regprecise | 24 | twwkkYttGaCYaktyytGaCCaa | 11.2163 | 0.467345833 | 1.70E-05 | 10.6 |
| CysR | SMU_852 | Transcriptional regulator of cysteine metabolism | Ref [79] | 13 | TATCASmGyGaTa | 10.8424 | 0.834030769 | 4.12E-05 | 8 |
| DnaA | SMU_01 | Chromosomal replication initiator protein | DBTBS (BS, DnaA, 44%, 5e-128, AAA, Bac_DnaA_C) | 9 | ttwtCcACA | -- | -- | DP | DP |
| FruR | SMU_870 | Transcriptional repressor of the fructose operon | Regprecise | 10 | tTGmtwGamw | 6.17237 | 0.617237 | 4.7E-05 | 8.4 |
| FurR | SMU_593 | Putative ferric uptake regulator protein | Regprecise | 15 | TtakAatsatTmTaa | 9.98815 | 0.665876667 | 5.76E-05 | 8.9 |
| GalR | SMU_885 | Galactose operon repressor | Regprecise | 19 | aaaatTTTASTAAAatttt | 11.0203 | 0.580015789 | 1.62E-05 | 12.5 |
| GlnR | SMU_363 | Glutamine synthetase repressor | Regprecise | 19 | aTGTyAkrwaaymTrACAt | 13.4055 | 0.705552632 | 9.95E-05 | 5.9 |
| HomR | SMU_930c | Transcriptional regulator of homocysteine metabolism | Ref [79] | 15 | TaTaGCyaaCyaTCa | 13.2191 | 0.881273333 | 7.94E-06 | 10.1 |
| HrcA | SMU_80 | Heat inducible transcription repressor | Regprecise | 27 | TTAGCaSTCkwkkdamaaGAGTGCTAA | 22.2571 | 0.824338 | 1.91E-05 | 5.9 |
| HtrA | SMU_2165c | HtrA transcription factor | DBTBS (BS, HtrA,38%, 3E-75, PDZ) | 8 | TTTYCaCa | -- | -- | DP | DP |
| LacR | SMU_1498 | Lactose repressor | Regprecise | 9 | aaaCaaaaa | -- | -- | DP | DP |
| LevR | SMU_1964c | Two component response regulator | DBTBS (BS, Des,31%,6E-28,REC,HTH_LUXR) | 17 | aTTTTtymkwwmwkyrt | -- | -- | DP | DP |
| MalR | SMU_1566 | Putative maltose operon transcriptional repressor | Regprecise | 20 | ttayGCAArCGyTTGCrywa | 15.3079 | 0.765395 | 3.27E-05 | 10 |
| MbrC | SMU_1008 | Cell envelope stress response regulator | Ref [54] | 14 | TTaCamtttTGTaa | 11.074 | 0.791 | 1.38E-05 | 12.1 |
| MleR | SMU_135c | Regulator of malolactic fermentation | DBTBS (BS, HexR, 22%, 3E-10) | 15 | aTCTaRyawaaKRtG | -- | -- | DP | DP |
| Mta | SMU_1790c | MerR family transcriptional regulator | DBTBS (BS, Mta, 37%, 7E-44,HTH_MERR) | 23 | GaCYCtMmCSymSSGtyatrGsC | 14.3604 | 0.624364 | 2.08E-06 | 9.4 |
| MtaR | SMU_1225 | Putative transcriptional regulator of methionine metabolism | Regprecise | 17 | tATAGtttmaarCTATA | 12.2283 | 0.719311765 | 6.03E-05 | 7.5 |
| NagR | SMU_1065c | GntR family transcriptional regulator of N acetylglucosamine utilization | Regprecise | 20 | maatwGGwMTAtACCawttw | 13.5623 | 0.678115 | 1.84E-05 | 9.1 |
| NmlR | SMU_677 | MerR family transcriptional regulator involved in carbonyl stress response | Regprecise | 19 | vCTTGaCTTGRaGTYvaCT | 16.8334 | 0.885968421 | 8.12E-06 | 8.3 |
| NrdR | SMU_1923c | Transcriptional regulator of ribonucleotide metabolism | Regprecise | 16 | aCaCaAtATmTtGtGt | 11.4352 | 0.7147 | 1.60E-05 | 10.9 |
| PdxR | SMU_953c | Putative transcriptional regulator of pyridoxine biosynthesis | Regprecise | NA° | TATTGTA-(18)-TACAATA | -- | -- | DP | DP |
| VicR | SMU_1517c | Two component response regulator | DBTBS (BS, YycF, 71%, 4E-114, REC, Trans_reg_C) | 17 | TGTamTrryGmYGkmak | 9.17903 | 0.539943 | 1.41E-05 | 9.7 |
| PflR | SMU_491 | Putative transcriptional regulator of formate metabolism | Regprecise | 19 | MGwAAywrawtyaaTTwCG | 11.7093 | 0.616278947 | 2.03E-05 | 8.4 |
| PipR | SMU_2134 | Putative transcriptional regulator | Regprecise | 19 | TaTraTmtAtTwtAtYatA | 12.1798 | 0.641042105 | 9.34E-05 | 7.5 |
| PurR | SMU_356 | Purine operon repressor | DBTBS (BS, PurR, 51%, 2E-96) | 8 | aaarCGaa | -- | -- | DP | DP |
| PyrR | SMU_856 | Pyrimidine operon regulatory protein | DBTBS (BS, PyrR, 54%, 1E-63) | 31 | CCTTTaaCmCTGTCCCGTGaGGCaGGCaaGG | 35.6892 | 1.15127 | 2.17E-06 | 16.7 |
| Rex | SMU_1053 | Redox-sensing transcriptional repressor | DBTBS (BS, Rex, 45%, 3e-62, CoA_binding) | 22 | TTaGYSwMTTTYTYwwCwaaRR | 15.6870 | 0.713044 | 7.84E-05 | 6.3 |
| RpoD | SMU_822 | RNA polymerase sigma-70 factor | DBTBS (BS, SigB, 25%, 1E-14) | NA° | ttGACa-N17-tatRat | -- | -- | DP | DP |
| ScrR | SMU_1844 | Sucrose operon repressor | Regprecise | 20 | tmtGyCAarCGytTGrCaka | 15.5795 | 0.778975 | 1.23E-05 | 9.5 |
| SdpR | SMU_1647c | Predicted transcriptional regulator | DBTBS (BS, SdpR, 51%, 7E-27, HTH_ARSR) | 7 | TCTAAAT | -- | -- | DP | DP |
| SloR | SMU_186 | Metal dependent transcriptional regulator | Ref [25,70] | 22 | maaagamwsytksSttatTtkS | 8.47192 | 0.385087 | 8.46E-05 | 8.2 |
| SMU_1193 | SMU_1193 | Putative transcriptional regulator of transporter genes | Regprecise | 22 | tTGTayyAtataawtaRtACAa | 13.9978 | 0.636263636 | 3.10E-05 | 8.9 |
| SMU_1349 | SMU_1349 | TetR family trancriptinal regulator | Ref [71] | 16 | TatTSTtTwwTRwTwa | -- | -- | DP | DP |
| SMU_2125 | SMU_2125 | Predicted transcriptional regulator | DBTBS (BS, HxlR, 36%, 8E-17) | 14 | CaCCyayvrTTTTt | -- | -- | DP | DP |
| SMU_289 | SMU_289 | Putative transcriptional regulator of ascorbate utilization genes | Regprecise | 22 | tTGaaCaCadrMSTGTGtKwYa | 15.8219 | 0.719178 | 1.03E-05 | 8.6 |
| TreR | SMU_2040 | Repressor of the trehalose operon | Regprecise | 20 | MMaaStTGYmkrCaaStTKK | 13.2721 | 0.663605 | 4.08E-05 | 8 |
|  |  |  |  |  |  |  |  |  |  |
