# Supplementary material for: Construction and verification of the transcriptional regulatory response network of Streptococcus mutans upon treatment with the biofilm inhibitor carolacton
Source: BMC Genomics. 2014 May 12;15:362. doi: 10.1186/1471-2164-15-362 (PMC4048456; doi:10.1186/1471-2164-15-362)
Supplement: Supplementary file 6 — Additional file 6: The different types of local network motifs identified in the S. mutans TRRN upon carolacton treatment. The motifs were identified and their corresponding statistical scores determined using the MFINDER tool [47, 119] after 10000 randomizations. *SD denotes the standard deviation of the number of occurrences of the motif in randomized networks. ^The “Z score” is a measure of the statistical significance of the motifs and is determined as (Mreal - Mrand)/SD. Motifs with P-values < = 0.01 were considered as significant. (DOCX 46 KB) [file 12864_2013_6097_MOESM6_ESM.docx]

| **The different types of local network motifs identified in the S.mutans TRRN upon carolacton treatment** | | | | |
| --- | --- | --- | --- | --- |
| **Network Motif** | **Common name** | **Number of occurrences**  **in the real network (M_real_)** | **Number of occurrences**  **in the randomized°**  **network (M_rand_) +/- SD*** | **Z score of motif occurrence^** |
| **** | **Feed forward loop (FFL)** | **11** | **2.5 +- 1.8** | **4.72** |
| **** | **Bi-fan motif** | **55** | **9.5 +- 4.3** | **10.58** |
| **** | **Multiple input module (MIM)** | **3** | **2.6 +- 0.6** | **0.67** |
| **** | **--** | **12** | **2.8 +- 2.6** | **3.54** |
| **** | **Possible bi-fan derivative** | **17** | **1.3 +- 2.1** | **7.48** |
| **** | **--** | **162** | **39.6 +- 27** | **4.53** |
| **** | **--** | **401** | **153 +- 133** | **1.86** |
