# Supplementary material for: Construction and verification of the transcriptional regulatory response network of Streptococcus mutans upon treatment with the biofilm inhibitor carolacton
Source: BMC Genomics. 2014 May 12;15:362. doi: 10.1186/1471-2164-15-362 (PMC4048456; doi:10.1186/1471-2164-15-362)
Supplement: Supplementary file 7 — Additional file 7: List of enriched categories in the co-regulated gene groups or subnetworks of the S. mutans TRRN upon carolacton treatment. Over-represented categories include KEGG metabolic pathways (KEGG MP), Biological functional classes (BFC) and gene ontology terms (GO). The details pertaining to the calculation of the significance scores are provided under the Methods section. (DOCX 58 KB) [file 12864_2013_6097_MOESM7_ESM.docx]

| **List of enriched categories in the co-regulated gene groups of the S.mutans TRRN upon carolacton treatment** | | | | | | | | |
| --- | --- | --- | --- | --- | --- | --- | --- | --- |
| **Enriched category term** | **Category type** | **Co-regulated group(CG)** | **Number of genes in the category [A]** | **Number of genes in the co-regulated groups [B]** | **Intersection**  **(A Π B)** | **P-value of Intersection** | **E- value of Intersection** | **Significance score** |
| Oxidative phosphorylation | KEGG MP | ctsR CG | 10 | 11 | 8 | 1.70E-15 | 3.84E-12 | 11.42 |
| Ribosome | KEGG MP | SMU.2125 CG | 49 | 14 | 9 | 8.40E-09 | 1.90E-05 | 4.72 |
| Pyrimidine metabolism | KEGG MP | pyrR CG | 40 | 9 | 6 | 7.60E-07 | 0.00172 | 2.76 |
| Valine leucine and isoleucine biosynthesis | KEGG MP | codY CG | 19 | 84 | 9 | 3.60E-05 | 0.08243 | 1.08 |
| Galactose metabolism | KEGG MP | galR CG | 18 | 7 | 3 | 0.00031 | 0.69901 | 0.16 |
| Nitrogen metabolism | KEGG MP | glnR CG | 10 | 13 | 3 | 0.00037 | 0.83221 | 0.08 |
| Purines pyrimidines nucleosides and nucleotides | BFC | pyrR CG | 61 | 9 | 8 | 5.20E-12 | 2.60E-09 | 8.58 |
| Protein synthesis | BFC | SMU.2125 CG | 123 | 14 | 9 | 1.90E-08 | 9.60E-06 | 5.02 |
| Amino acid biosynthesis | BFC | codY CG | 86 | 84 | 17 | 4.60E-08 | 2.40E-05 | 4.63 |
| Energy metabolism | BFC | ctsR CG | 149 | 11 | 8 | 1.30E-07 | 6.90E-05 | 4.16 |
| Cell envelope | BFC | mbrC CG | 81 | 4 | 3 | 0.00027 | 0.13868 | 0.86 |
| Transport and binding proteins | BFC | SMU.1193 CG | 210 | 14 | 7 | 0.00027 | 0.14075 | 0.85 |
| Signal transduction | BFC | ccpA CG | 61 | 22 | 5 | 0.00045 | 0.23044 | 0.64 |
| Transport and binding proteins | BFC | homR CG | 210 | 5 | 4 | 0.00061 | 0.3109 | 0.51 |
| Transport and binding proteins | BFC | ccpA CG | 210 | 22 | 8 | 0.00135 | 0.6938 | 0.16 |
| Amino acid biosynthesis | BFC | glnR CG | 86 | 13 | 4 | 0.00188 | 0.96285 | 0.02 |
| Hydrogen ion transporting ATPase activity, rotational mechanism | GO | ctsR CG | 8 | 11 | 8 | 3.50E-17 | 4.50E-13 | 12.35 |
| Hydrogen ion transporting ATP synthase activity, rotational mechanism | GO | ctsR CG | 8 | 11 | 8 | 3.50E-17 | 4.50E-13 | 12.35 |
| Ribosome | GO | SMU.2125 CG | 48 | 14 | 9 | 6.40E-09 | 8.10E-05 | 4.09 |
| Structural constituent of ribosome | GO | SMU.2125 CG | 49 | 14 | 9 | 7.80E-09 | 9.90E-05 | 4 |
| Translation | GO | SMU.2125 CG | 55 | 14 | 9 | 2.30E-08 | 0.0003 | 3.53 |
| Intracellular | GO | SMU.2125 CG | 69 | 14 | 8 | 3.50E-06 | 0.0449 | 1.35 |
