# Supplementary figures and images for: Construction and verification of the transcriptional regulatory response network of Streptococcus mutans upon treatment with the biofilm inhibitor carolacton
Source: BMC Genomics. 2014 May 12;15:362. doi: 10.1186/1471-2164-15-362 (PMC4048456; doi:10.1186/1471-2164-15-362)

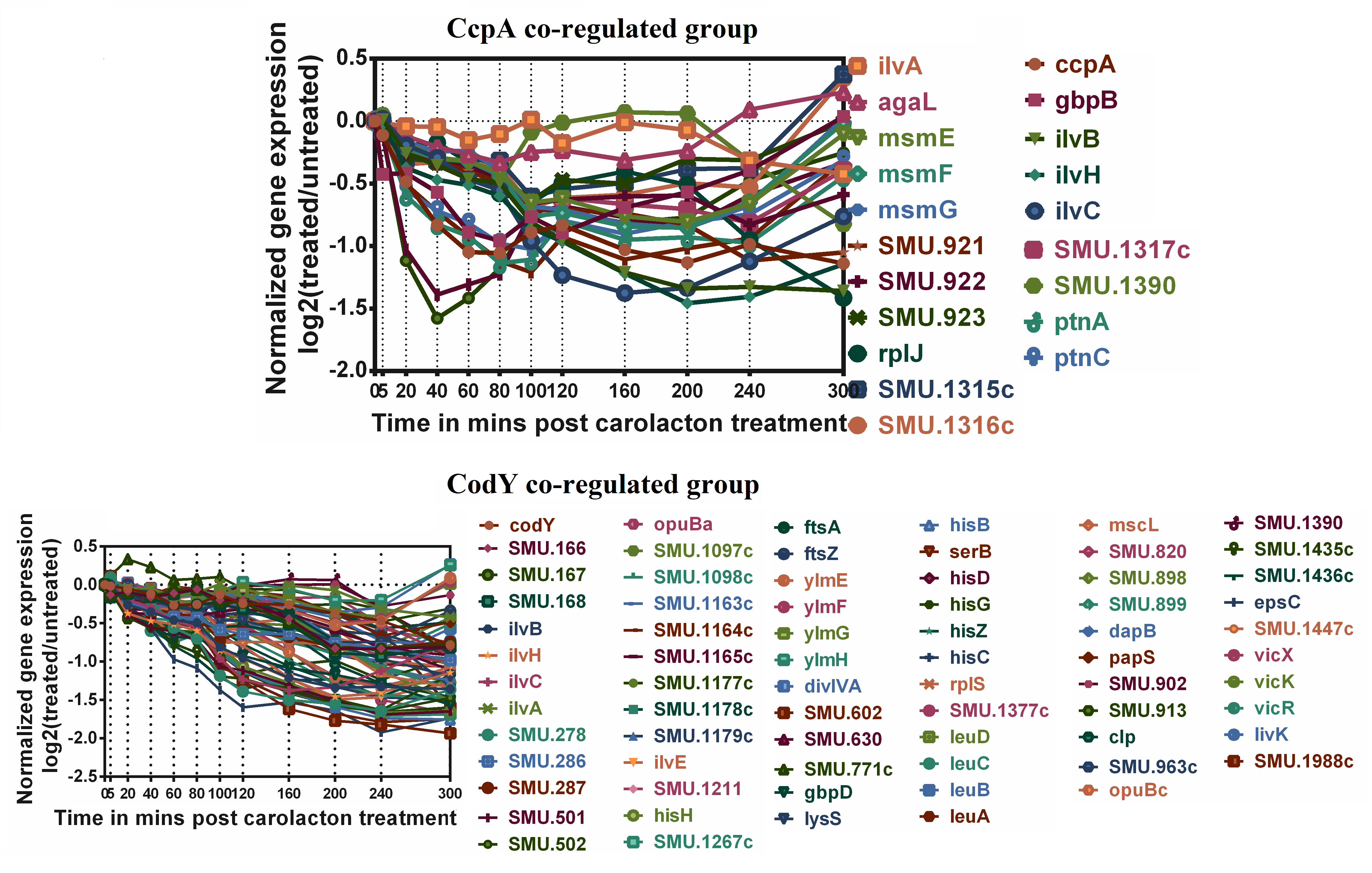

Supplement: Supplementary file 8 — Additional file 8: The normalized expression profiles of the genes co-regulated by the global transcription factors CcpA and CodY. For the CodY co-regulated gene group, only the expression profiles of genes with positive relationships are shown. (PNG 755 KB) [file 12864_2013_6097_MOESM8_ESM.png]
