# Supplementary material for: Construction and verification of the transcriptional regulatory response network of Streptococcus mutans upon treatment with the biofilm inhibitor carolacton
Source: BMC Genomics. 2014 May 12;15:362. doi: 10.1186/1471-2164-15-362 (PMC4048456; doi:10.1186/1471-2164-15-362)
Supplement: Supplementary file 9 — Additional file 9: Primers used in the study. (DOCX 36 KB) [file 12864_2013_6097_MOESM9_ESM.docx]

| **Primers used for the PCR-amplification of the coding sequences of the response regulators MbrC and VicR of *S.mutans* as well as for the amplification of the potential MbrC binding sites in the promoter regions of known and predicted targets** | | |
| --- | --- | --- |
| **Primer** | **Nucleotide sequence (5`- 3`)** | **Purpose** |
| MbrCFor | taagaaggagatataccatggctatgctaaagcaagaaaaaatttac | Expression of MbrC |
| MbrCRev | gtggtggtggtggtgctcgagtttaattaaataccctactcctctt | Expression of MbrC |
| VicRFor | \| taagaaggagatataccatggctatgaagaaaattctaatcgttgacg \| \| --- \| | Expression of VicR |
| VicRRev | \| gtggtggtggtggtgctcgaggtcatatgatttcatgtaataacca \| \| --- \| | Expression of VicR |
| EM1006F | gagctgtaaatttctcaggcttct | Forward primer for amplification of MbrC binding site in the SMU_1006 promoter (positive control) |
| EM1006R | ttgatttctaaaaacatagcttctcc | Reverse primer for amplification of MbrC binding site in the SMU_1006 promoter (positive control) |
| EM610F | tttgtgctttagaattaatgttgga | Forward primer for amplification of potential MbrC binding site in the SMU_610 promoter |
| EM610R | ttttacttttacgaaaaccgtaagtt | Reverse primer for amplification of potential MbrC binding site in the SMU_610 promoter |
| EM718F | ttgcaagcaagcaataaatatga | Forward primer for amplification of potential MbrC binding site in the SMU_718 promoter |
| EM718R | \| ttagcatcttggatagcagtgta \| \| --- \| | Reverse primer for amplification of potential MbrC binding site in the SMU_718 promoter |

| **Primers used for the deletion of MbrC binding sites** | | |
| --- | --- | --- |
| **Primer** | **Nucleotide sequence (5`- 3`)** | **Purpose** |
| 610Del1 | tttgtaatataaattgatttgatttgtcaaattttttgcttg | Deletion of potential MbrC site in SMU_610 promoter |
| 610Del2 | tcaaatcaatttatattacaaaaatatatttggaagattta | Deletion of potential MbrC site in SMU_610 promoter |
| 718Del1 | ttcctaatttctctatcttactaaaaattagcttatttgtc | Deletion of potential MbrC site in SMU_718 promoter |
| 718Del2 | tagtaagatagagaaattaggaaccagtatgtcaatta | Deletion of potential MbrC site in SMU_718 promoter |
| 1006Del1 | ttaatgtcaattacgattctttaagtg | Deletion of MbrC site in SMU_1006 promoter |
| 1006Del2 | aaagaatcgtaattgacattaatttaat | Deletion of MbrC site in SMU_1006 promoter |

| **Primers used for constructing the gene deletion mutants** | | |
| --- | --- | --- |
| **Primer** | **Sequence** | **Function** |
| CysR-P1 | TGTCAAACGCGAGGTGTTAG | *cysR* deletion |
| CysR-P2 | GGCGCGCCCGAAAGGTACCGCTGTTAGC | *cysR* deletion |
| CysR-P3 | GGCCGGCCCTTATCCACGTGTGCTCTCAAA | *cysR* deletion |
| CysR-P4 | GCCTGATGTGCTTGATCATTT | *cysR* deletion |
| FabT-P1 | TTCGCATTGCAGAACTTGTC | *fabT* deletion |
| FabT-P2 | GGCGCGCCGTTTTCAAGCTCATCTCTTCGAT | *fabT* deletion |
| FabT-P3 | GGCCGGCCTGGTGCTTTGGAGAAGGGGTTA | *fabT* deletion |
| FabT-P4 | TTCCATTTTCGACACATTCG | *fabT* deletion |
| Rgg-P1 | CACGTAAGAGCAACAATCTAGCC | *rgg* deletion |
| Rgg-P2 | GGCGCGCCTTCATTTTTAGACCGCGTGA | *rgg* deletion |
| Rgg-P3 | GGCCGGCCAGCAATGAAATTGGCAGGGAGT | *rgg* deletion |
| Rgg-P4 | GACATCACTTTCATTTGGAGGA | *rgg* deletion |
| SpxA-P1 | TGGGCTTCATAGAGGGCATA | *spxA* deletion |
| SpxA-P2 | GGCGCGCCCTCGCTTTACGGCAGCTTGTA | *spxA* deletion |
| SpxA-P3 | GGCCGGCCTCGCAAACAAGAACTGCGTCAA | *spxA* deletion |
| SpxA-P4 | CAGCAGCATAGTCCCAAGGT | *spxA* deletion |
| GlnR-P1 | TAGCAAAGGGGTGGATTTGT | *glnR* deletion |
| GlnR-P2 | GGCGCGCCATAGGAAAAACCGCCATTGA | *glnR* deletion |
| GlnR-P3 | GGCCGGCCACGCAACATCTTGGTGGTTTA | *glnR* deletion |
| GlnR-P4 | GGAGCTTCATAACCAGGAACC | *glnR* deletion |
| C-CysR1-F | GTGGCAGGTTATGTTAATAAAGGCGAA | Complementation *cysR* |
| C-CysR1-R | CCTAGACGAATATATTTGACAACAAAAAACTTG | Complementation *cysR* |
| Q-CysR1-F | CCTTTGCTGACTGCCTTTTC | Quantitative RT-PCR |
| Q-CysR1-R | GTGTCCTTGGGCAACTTCAT | Quantitative RT-PCR |
| Q-CysR2-F | CTTTGCTGACTGCCTTTTCC | Quantitative RT-PCR |
| Q-CysR2-R | TCGGTCGGTCACATTGTAAA | Quantitative RT-PCR |
| Q-CysR3-F | TTGTCAGTCAGCCCAGTTTG | Quantitative RT-PCR |
| Q-CysR3-R | TCGGTCGGTCACATTGTAAA | Quantitative RT-PCR |
| ComE-F | GCGATGGTCGACAATTATATAATCAATTGACAACGGC | RT-PCR positive control |
| ComE-R | GCGATGGTCGACTCATTTTGCTCTCCTTTGATCAG | RT-PCR positive control |
| ComX-F | ATTCCGGCATAGCTCAGTTG | RT-PCR positive control |
| ComX-R | GCGATGCATATGTTTTAGCCGGAGCTTTTTCA | RT-PCR positive control |
